# Unraveling a Tangled Skein: Evolutionary Analysis of the Bacterial Gibberellin Biosynthetic Operon

**DOI:** 10.1128/mSphere.00292-20

**Published:** 2020-06-03

**Authors:** Ryan S. Nett, Huy Nguyen, Raimund Nagel, Ariana Marcassa, Trevor C. Charles, Iddo Friedberg, Reuben J. Peters

**Affiliations:** a Roy J. Carver Department of Biochemistry, Biophysics & Molecular Biology, Iowa State University, Ames, Iowa, USA; b Department of Veterinary Microbiology and Preventive Medicine, Iowa State University, Ames, Iowa, USA; c Department of Biology, University of Waterloo, Waterloo, Ontario, Canada; University of Illinois at Urbana—Champaign

**Keywords:** gibberellin, operon evolution, plant-microbe interactions

## Abstract

Gibberellin (GA) phytohormones are ubiquitous regulators of growth and developmental processes in vascular plants. The convergent evolution of GA production by plant-associated bacteria, including both symbiotic nitrogen-fixing rhizobia and phytopathogens, suggests that manipulation of GA signaling is a powerful mechanism for microbes to gain an advantage in these interactions. Although orthologous operons encode GA biosynthetic enzymes in both rhizobia and phytopathogens, notable genetic heterogeneity and scattered operon distribution in these lineages, including loss of the gene for the final biosynthetic step in most rhizobia, suggest varied functions for GA in these distinct plant-microbe interactions. Therefore, deciphering GA operon evolutionary history should provide crucial evidence toward understanding the distinct biological roles for bacterial GA production. To further establish the genetic composition of the GA operon, two operon-associated genes that exhibit limited distribution among rhizobia were biochemically characterized, verifying their roles in GA biosynthesis. This enabled employment of a maximum parsimony ancestral gene block reconstruction algorithm to characterize loss, gain, and horizontal gene transfer (HGT) of GA operon genes within alphaproteobacterial rhizobia, which exhibit the most heterogeneity among the bacteria containing this biosynthetic gene cluster. Collectively, this evolutionary analysis reveals a complex history for HGT of the entire GA operon, as well as the individual genes therein, and ultimately provides a basis for linking genetic content to bacterial GA functions in diverse plant-microbe interactions, including insight into the subtleties of the coevolving molecular interactions between rhizobia and their leguminous host plants.

**IMPORTANCE** While production of phytohormones by plant-associated microbes has long been appreciated, identification of the gibberellin (GA) biosynthetic operon in plant-associated bacteria has revealed surprising genetic heterogeneity. Notably, this heterogeneity seems to be associated with the lifestyle of the microbe; while the GA operon in phytopathogenic bacteria does not seem to vary to any significant degree, thus enabling production of bioactive GA, symbiotic rhizobia exhibit a number of GA operon gene loss and gain events. This suggests that a unique set of selective pressures are exerted on this biosynthetic gene cluster in rhizobia. Through analysis of the evolutionary history of the GA operon in alphaproteobacterial rhizobia, which display substantial diversity in their GA operon structure and gene content, we provide insight into the effect of lifestyle and host interactions on the production of this phytohormone by plant-associated bacteria.

## Abstract (precis)

While production of phytohormones by plant-associated microbes has long been appreciated, identification of the gibberellin (GA) biosynthetic operon in plant-associated bacteria has revealed surprising genetic heterogeneity. Notably, this heterogeneity seems to be associated with the lifestyle of the microbe; while the GA operon in phytopathogenic bacteria does not seem to vary to any significant degree, thus enabling production of bioactive GA, symbiotic rhizobia exhibit a number of GA operon gene loss and gain events. This suggests that a unique set of selective pressures are exerted on this biosynthetic gene cluster in rhizobia. Through analysis of the evolutionary history of the GA operon in alphaproteobacterial rhizobia, which display substantial diversity in their GA operon structure and gene content, we provide insight into the effect of lifestyle and host interactions on the production of this phytohormone by plant-associated bacteria.

## INTRODUCTION

The clustering of bacterial biosynthetic genes within operons allows for the controlled coexpression of functionally related genes under a single promoter and the opportunity for these genes to be mobilized and coinherited as a complete metabolic unit via horizontal gene transfer (HGT) (1, 2). Because operon-localized genes are responsible for many fundamental biosynthetic pathways in bacteria, analysis of the genetic structure of complex operons can provide important clues regarding the selective pressures driving the evolution of bacterial metabolism and can also yield insight into the occurrences and mechanisms of HGT.

**FIG 1:**
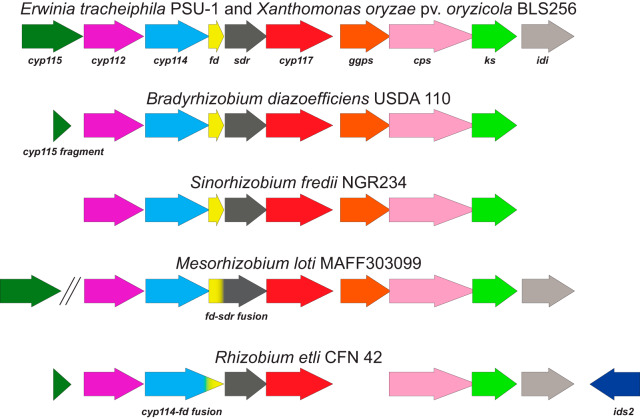
Diversity among GA biosynthetic operons in different bacterial lineages. The core operon genes are defined as *cyp112*, *cyp114*, *fd*, *sdr*, *cyp117*, *ggps*, *cps*, and *ks*, as these are almost always present within the GA operon. Other genes, including *cyp115*, *idi*, and *ids2*, exhibit a more limited distribution among GA operon-containing species. The double slanted lines in the Mesorhizobium loti MAFF303099 operon indicate that *cyp115* is not located adjacent to the rest of the operon.

The ability of bacteria to produce gibberellin (GA), a major plant hormone, is imparted by a GA biosynthetic operon (GA operon) (Fig. 1), which is found in both nitrogen-fixing rhizobia and phytopathogenic bacteria (3–5). While the diterpenoid GA phytohormones act as endogenous signaling molecules for growth and development in vascular plants (6), plant-associated fungi and bacteria have convergently evolved the ability to produce GA as a mechanism for host manipulation (4, 7–9). The phenomenon of GA production by plant-associated microbes has important biological implications, as perturbation in GA signaling can lead to extreme phenotypic changes in plants. For example, production of GA by the rice pathogen Gibberella fujikuroi leads to dramatic elongation and eventual lodging of rice crops (10), and impaired GA metabolism is responsible for the semidwarf crop phenotypes associated with crops utilized within the Green Revolution (11, 12). More recently, it has been shown that GA acts as a virulence factor for phytopathogenic bacteria (9) and can affect nodulation phenotypes when produced by rhizobia in symbiosis with legumes (4). Therefore, studying the biosynthesis and biological function of microbial GA is crucial to our understanding of how these plant-microbe interactions can affect plant health and development.

The GA operon was discovered in the rhizobial symbiont of soybean, Bradyrhizobium diazoefficiens (formerly *B. japonicum*) USDA 110 (13). This operon contains a geranylgeranyl diphosphate synthase (*ggps*), two diterpene synthases/cyclases (*cps* and *ks*), three cytochrome P450 (CYP) monooxygenases (*cyp112*, *cyp114*, and *cyp117*), a short-chain dehydrogenase/reductase (*sdr*_GA_), and a ferredoxin (*fd*_GA_) (13, 14). The *B. diazoefficiens* operon also contains a severely truncated presumably nonfunctional CYP gene (pseudo *cyp115* [p-*cyp115*]) located at the 5′ end of the operon. The core gene cluster, which contains all of the aforementioned genes other than *cyp115*, is widely distributed within symbiotic nitrogen-fixing rhizobia from the *Alphaproteobacteria* class (here referred to as α-rhizobia for simplicity; see Materials and Methods for definition) (15). Biochemical characterization of GA operon genes in several α-rhizobia, including *B. diazoefficiens*, Sinorhizobium fredii, and Mesorhizobium loti, has demonstrated that this core operon is responsible for biosynthesis of GA_9_, the penultimate intermediate to the bioactive phytohormone GA_4_ (3, 4, 16–18). While seemingly only found in plant-associated bacteria (19), the GA operon exhibits scattered distribution within the α-rhizobia, and functional versions of the operon can also be found in several *Betaproteobacteria* rhizobial symbionts (here referred to as β-rhizobia; see Materials and Methods for definition) (20, 21). Analogous GA operons can be found in certain gammaproteobacterial plant pathogens as well (e.g., *Xanthomonas* and *Erwinia* species), and characterization of the GA operon from several distant gammaproteobacterial lineages has demonstrated that the biosynthetic functionality of this operon is conserved (5, 9, 20).

The abundance of sequenced bacterial genomes indicates that the GA operon structure is more complex and variable than that initially described for *B. diazoefficiens*, the species in which this operon was initially identified (14, 15). Specifically, certain bacteria with the GA operon were found to contain a full-length *cyp115* gene at the 5′ end of the gene cluster as opposed to a pseudogene/fragment. This enzyme (CYP115) has been shown to catalyze the final step in bioactive GA biosynthesis, converting GA_9_ into bioactive GA_4_ (5, 20, 22). Additionally, many bacterial strains possess a putative isopentenyl diphosphate δ-isomerase (*idi*) gene located at the 3′ end of the operon, which presumably functions in balancing the concentrations of the (di)terpenoid building blocks, isopentenyl diphosphate (IPP) and dimethylallyl diphosphate (DMAPP) (23). Full-length *cyp115* and *idi* genes are notably absent from many α- and β-rhizobia with the operon, while copies of these genes are essentially always present in the GA operons of gammaproteobacterial phytopathogens (Fig. 1). Intriguingly, it appears that some of the α-rhizobia have specifically lost these genes, as fragments of both *cyp115* and *idi* can be found flanking the core gene cluster in many of the relevant species/strains (22, 24). Moreover, a small number of α-rhizobia have a presumably inactivating frameshift mutation in the canonical *ggps* within their operon (17) but also have an additional isoprenyl diphosphate synthase (IDS) gene adjacent to the operon (*ids2*), which could potentially compensate for the loss of *ggps*. Collectively, this heterogeneity of the GA operon in rhizobia provides an excellent opportunity for analyzing the formation and reorganization of bacterial gene clusters.

Initial phylogenetic analyses of the GA operon suggested that it may have undergone HGT among bacterial lineages (17, 20). Furthermore, the varied genetic structures of the operon in divergent species, including both symbionts and pathogens, suggest that selective pressures unique to certain bacteria may be driving the acquisition or loss of not only the GA operon but also some of the associated genes. Thus, detailed analysis of GA operon evolution will help elucidate the evolutionary processes that have shaped bacterial GA biosynthesis in plant-microbe interactions. Here, the predicted biochemical functions were assessed and confirmed for the *idi* and *ids2* genes that are sporadically associated with the GA operon, thereby providing evidence for their roles in GA biosynthesis. This clarification of genetic content prompted further analysis of the distribution and function of the GA operon in bacteria more generally, thereby providing an overview of the genetic diversity and evolutionary history of this gene cluster. Using an algorithm developed to analyze the assembly and evolution of gene blocks (i.e., genes within operons/clusters) (25), the distribution and phylogeny of the GA operon was further analyzed within the α-rhizobia, as this lineage displays a notable amount of diversity in operon structure and genetic content. Altogether, this thorough assessment of the underlying genetics and biochemistry of the GA operon allows for the formulation of informed hypotheses regarding the biological function of GA production within diverse bacterial lineages.

## RESULTS

### Biochemical characterization of two accessory GA operon genes.

**FIG 2:**
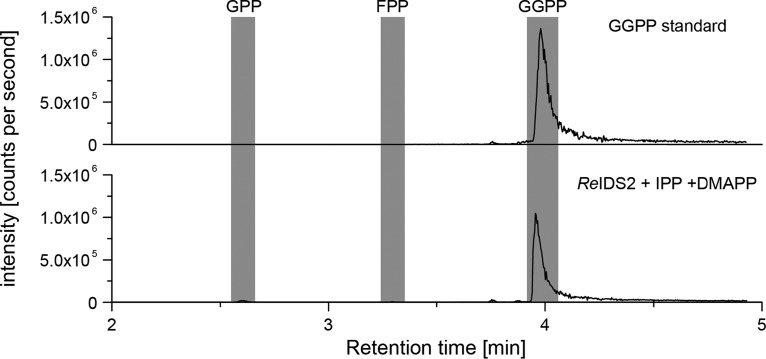
*In vitro* characterization of *Re*IDS2. LC-MS/MS chromatograms for the *in vitro* enzyme assay of *Re*IDS2 with IPP and DMAPP as the substrates (bottom) in comparison to an authentic GGPP standard (top). Note that trace amounts of geranyl diphosphate (GPP) and farnesyl diphosphate (FPP) were detected as products of the enzyme assay. Intensity of peaks is measured in ion counts per second.

The general diterpenoid precursor (*E*,*E*,*E*)-geranylgeranyl diphosphate (GGPP) is essential as a precursor for GA biosynthesis. Unlike plants, which require GGPP for the biosynthesis of chlorophyll (26), diterpenoid biosynthesis (and thus GGPP production) does not appear to be ubiquitous among bacteria (27). Therefore, a functional *ggps* gene must be maintained along with the rest of the GA operon for GA biosynthesis to occur. In the GA operon-containing strain Rhizobium etli CFN 42, the operon *ggps* contains a frameshift mutation that results in a severely truncated protein (17). However, a second predicted IDS gene (*ids2*) with low sequence identity to the canonical operon *ggps* found in other *Rhizobium* species (≤30% amino acid sequence identity) is found in close proximity to this strain’s operon (Fig. 1). Closely related homologs of *ids2* are similarly situated nearby in a number of other α-rhizobia with GA operons wherein *ggps* also appears to be inactive (see Table S1 in the supplemental material). Given the conservation of these modified operons, we hypothesized that the encoded IDS2 enzyme also catalyzes the formation of GGPP, thereby restoring functionality to these GA operons. Indeed, recombinantly expressed and purified IDS2 protein from *R. etli* CFN 42 (*Re*IDS2) produced GGPP as its major product from the universal isoprenoid precursors IPP and DMAPP (Fig. 2). Thus, IDS2 can functionally complement the loss of the canonical GGPS enzyme to restore production of GA in these operons. Accordingly, here, these *ids2* gene orthologs are referred to as *ggps2* to reflect their biochemical function (e.g., *Re*IDS2 becomes *Re*GGPS2).

10.1128/mSphere.00292-20.6 TABLE S1 Pairwise alignments of GGPS2 proteins and representative GGPS proteins. Download Table S1, DOCX file, 0.1 MB. Copyright © 2020 Nett et al. 2020 Nett et al. This content is distributed under the terms of the Creative Commons Attribution 4.0 International license.

**FIG 3:**
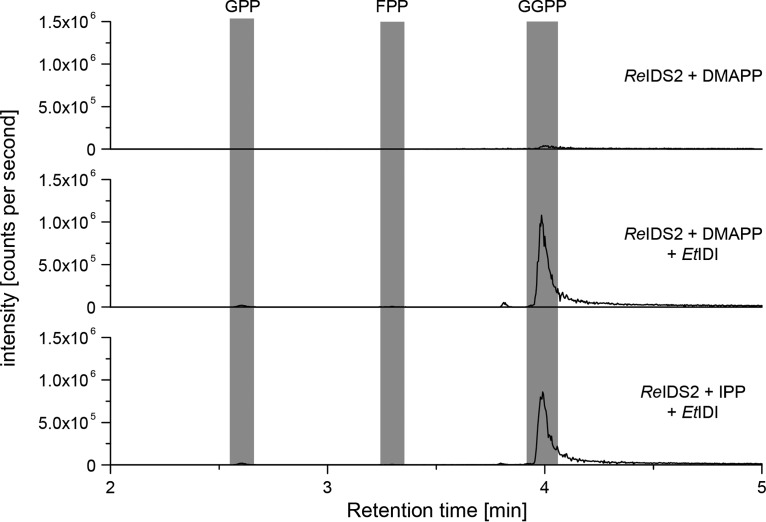
*In vitro* characterization of *Et*IDI. LC-MS/MS chromatograms for the *in vitro* enzyme assay of *Re*IDS2. Shown are results with DMAPP as the substrate (top), *Re*IDS2 and *Et*IDI combination assay with DMAPP as the substrate (middle), and *Re*IDS2 and *Et*IDI combination assay with IPP as the substrate (bottom). Intensity of peaks is measured in ion counts per second.

The only remaining gene strongly associated with the GA operon but not yet characterized was *idi*, which has been presumed to be involved in balancing the ratio of IPP and DMAPP isoprenoid building blocks for diterpenoid biosynthesis (23). Thus, the GA operon *idi* from Erwinia tracheiphila (*Et*IDI), a gammaproteobacterial plant pathogen, was cloned and heterologously expressed in Escherichia coli. To test for activity, a coupled enzyme assay with purified *Et*IDI and *Re*GGPS2 proteins was employed. Because IDS enzymes require both IPP and DMAPP as cosubstrates, *Re*GGPS2 is unable to produce GGPP when only IPP or DMAPP is supplied as the sole substrate. Addition of *Et*IDI to these reactions enabled the production of GGPP by *Re*GGPS2 in the presence of either IPP or DMAPP alone (Fig. 3), thus indicating that *Et*IDI can effectively interconvert these two substrates.

### HGT of the GA operon within alphaproteobacterial rhizobia.

The scattered distribution of the GA operon among three classes of proteobacteria suggests HGT of this gene cluster. A previous phylogenetic analysis suggests that the ancestral gene cluster initially evolved within gammaproteobacterial phytopathogens, as their operon genes exhibit greater phylogenetic divergence than those in the rhizobia, and that the operon was subsequently acquired by α- and β-rhizobia in separate HGT events (20). Additionally, specific phylogenetic analysis of the GA operon within α-rhizobia suggests that it may have subsequently undergone further HGT within this class (17).

**FIG 4:**
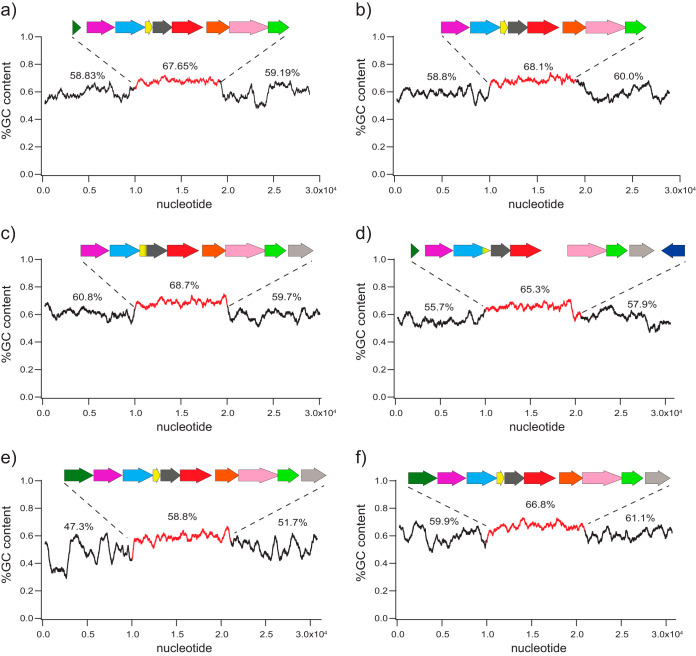
GC content analysis of the GA operon in a range of bacterial lineages. Shown are the average percent GC contents within 500-bp windows for the GA operon (red) and flanking 10-kb regions for the following species: (a) B. diazoefficiens USDA 110, (b) *S. fredii* NGR234, (c) M. loti MAFF303099, (d) *R. etli* CFN 42, (e) *E. tracheiphila* PSU-1, and (f) *X. oryzae* pv. *oryzicola* BLS256. Shown above each region is the average percent GC content.

It was previously noted that the GC content of the GA operon in rhizobia is particularly high compared with that of the surrounding genomic sequence (14, 24, 28, 29), a phenomenon that is often associated with HGT (30). To better assess the increased GC content of the GA operon, we analyzed the gene cluster sequences and the surrounding DNA in exemplary genomes from four of the major α-rhizobial genera (*Bradyrhizobium*, *Mesorhizobium*, *Rhizobium*, and *Sinorhizobium*) and two genera of the gammaproteobacterial plant pathogens (*Erwinia* and *Xanthomonas*). In each case, the GA operon has noticeably higher GC content than the surrounding DNA (≥7% higher), with sharp drops in GC content preceding and following the operon (Fig. 4). This elevated GC content is also observed within the remnants of GA operon genes such as *cyp115* and *idi* that have been lost in some strains; for example, an *idi* fragment with elevated GC content can be found 3′ to the *ks* gene in the *S. fredii* NGR234 operon (Fig. 4b). Further support for HGT of the GA operon has been suggested by the presence of insertional sequence (IS) elements flanking the operon (e.g., transposases and integrases) in many species (5, 22). Overall, these collective observations strongly support HGT of the GA operon, consistent with its widely scattered distribution throughout the proteobacteria. Interestingly, the *ggps2* gene associated with the *R. etli* CFN 42 operon exhibits markedly lower GC content (∼7% lower) than the rest of the operon (Fig. 4d). This also seems to be true for other strains containing *ggps2* and supports relatively recent acquisition of this gene by the core GA operon (see Table S2).

10.1128/mSphere.00292-20.7 TABLE S2 Percent GC content in the core GA operon (*cyp112*-*ks*) compared to that of *ggps2*. Download Table S2, DOCX file, 0.1 MB. Copyright © 2020 Nett et al. 2020 Nett et al. This content is distributed under the terms of the Creative Commons Attribution 4.0 International license.

### Gene cluster analysis.

While the GA operons found in *Gammaproteobacteria* exhibit essentially uniform gene content and structural composition, those from the α-rhizobia exhibit much more diversity in genetic structure. This suggests that selective pressures specific to the rhizobia, presumably their symbiotic relationship with legumes, may have driven this heterogeneity in the operon. To better understand the evolutionary history of the GA operon in the α-rhizobia, a more thorough analysis was carried out with Reconstruction of Ancestral Genomes Using Events (ROAGUE) software (25, 31). ROAGUE generates a phylogenetic tree with selected taxa that contain gene blocks (i.e., gene clusters) of interest and then uses a maximum parsimony approach to reconstruct a predicted gene block structure at each ancestral node of the tree. Using this ROAGUE approach, the evolutionary events involved in the genetic construction of orthologous GA operon gene blocks in the α-rhizobia, specifically, gene loss, gain, and duplication, were quantitatively assessed (see Fig. S1 for a summary of the method pipeline). A total of 118 α-rhizobia with at least a minimal set of GA operon genes were initially identified and included in this analysis (see Table S3 for a list of the strains). The most phylogenetically distant GA operon to those in the α-rhizobia is found within *E. tracheiphila* (20), and as such, this was used as an outgroup. Additionally, to observe the relative relationship between alpha- and gammaproteobacterial operons, the GA operon from Xanthomonas oryzae was also included in the analysis.

10.1128/mSphere.00292-20.1 FIG S1 Method pipeline for the ROAGUE-generated ancestral operon reconstructions. Download FIG S1, EPS file, 1.7 MB. Copyright © 2020 Nett et al. 2020 Nett et al. This content is distributed under the terms of the Creative Commons Attribution 4.0 International license.

10.1128/mSphere.00292-20.8 TABLE S3 List of alphaproteobacterial strains used within the ancestral reconstruction analyses. Download Table S3, DOCX file, 0.1 MB. Copyright © 2020 Nett et al. 2020 Nett et al. This content is distributed under the terms of the Creative Commons Attribution 4.0 International license.

An initial reconstruction was made by creating a species tree using the amino acid sequence of *rpoB* (RNA polymerase β subunit) from each strain as the phylogenetic marker gene (“full species tree” [FS]) (see Fig. S2). However, the species tree is rarely indicative of a given gene’s evolution and even less so concerning operon evolution where HGT is involved. To better understand the evolution of the GA operon in relationship to the bacterial species, we constructed a second tree with concatenated protein sequences comprising the core GA operon (“full operon tree” [FO]) (see Fig. S3). Due to the large number of species being analyzed, along with apparent phylogenetic redundancy that could introduce bias, reconstructions were also made with only more distinct representative strains by using the Phylogenetic Diversity Analyzer (PDA) program, which reduced the number of analyzed taxa to 64 (32). These reduced phylogenetic trees are referred to as the “partial species tree” (PS) (see Fig. S4) and the “partial operon tree” (PO) (see Fig. S5).

10.1128/mSphere.00292-20.2 FIG S2 Ancestral reconstruction of the GA biosynthetic operon using *rpoB* for phylogenetic analysis. A phylogenetic tree was constructed with alignments of *rpoB* protein sequences from 118 α-rhizobial species and two *Gammaproteobacteria* using the neighbor-joining method as a measure of distance between species. ROAGUE was then applied to create the ancestral operon reconstruction. The lowercase letters in each tree node represent the genes in the orthoblock (e.g., “a” represents “*cyp115*”), with each gene additionally indicated by a unique color (see legend at top). A blank space between genes designates a split of ≥500 bp between the genes to either side of the blank space. The green bar on the top left displays the total number of events that occur in this reconstruction. For each inner node *u*, the floating number (e.g., 98.0) represents the bootstrap value of the tree. The numbers in the brackets indicate the cumulative counts of events going from the leaf nodes to node *u* in the following order: deletions, duplications, and splits. Each leaf node is accompanied with symbols (*, ?, and !), the genomic accession number, the species/strain name, and the gene block for that strain. *, the gene block contains full-length *cyp115* (gene “a”); !, the gene block contains a truncation/fragment of *cyp115*; ?, the gene block contains *ggps2* (gene “k”). The reference strain, Xanthomonas oryzae pv. *oryzicola* BLS256, is in blue and the outgroup strain, Erwinia tracheiphila PSU-1, is in gray. These naming and color conventions persist throughout this study. Download FIG S2, PDF file, 2.3 MB. Copyright © 2020 Nett et al. 2020 Nett et al. This content is distributed under the terms of the Creative Commons Attribution 4.0 International license.

10.1128/mSphere.00292-20.3 FIG S3 Ancestral reconstruction of the GA biosynthetic operon using the concatenated operon for phylogenetic analysis. A phylogenetic tree was constructed with alignments of concatenated proteins from core GA operon genes (*cyp112-cyp114-fd-sdr-cyp117-cps-ks*) from 118 α-rhizobial species and two *Gammaproteobacteria* using the neighbor-joining method. ROAGUE was then applied to create the ancestral operon reconstruction. Fusion events between *cyp114*-*fd* and *fd*-*sdr* were determined manually and are indicated by hashtag (#) and dollar sign ($) symbols, respectively, next to the name of the relevant strain. All other annotations are described in the legend for Fig. S2. Download FIG S3, PDF file, 2.3 MB. Copyright © 2020 Nett et al. 2020 Nett et al. This content is distributed under the terms of the Creative Commons Attribution 4.0 International license.

10.1128/mSphere.00292-20.4 FIG S4 Reduced ancestral reconstruction of the GA biosynthetic operon using *rpoB* for phylogenetic analysis. A phylogenetic tree was constructed with alignments of *rpoB* protein sequences from 118 α-rhizobial species and two *Gammaproteobacteria* using the neighbor-joining method as a measure of distance between species. The number of analyzed species was reduced to 64 with the Phylogenetic Diversity Analyzer software, and ROAGUE was then applied to create the ancestral operon reconstruction. All other annotations are described in the legend for Fig. S2. Download FIG S4, PDF file, 1.3 MB. Copyright © 2020 Nett et al. 2020 Nett et al. This content is distributed under the terms of the Creative Commons Attribution 4.0 International license.

10.1128/mSphere.00292-20.5 FIG S5 Reduced ancestral reconstruction of the GA biosynthetic operon using the concatenated operon for phylogenetic analysis. A phylogenetic tree was constructed with alignments of concatenated proteins from core GA operon genes (*cyp112-cyp114-fd-sdr-cyp117-cps-ks*) from 118 α-rhizobial species and two *Gammaproteobacteria* using the neighbor-joining method. The number of analyzed species was reduced to 64 with the Phylogenetic Diversity Analyzer software, and ROAGUE was then applied to create the ancestral operon reconstruction. Fusion events between *cyp114*-*fd* and *fd*-*sdr* were determined manually and are indicated by hashtag (#) and dollar sign ($) symbols, respectively, next to the name of the relevant strain. All other annotations are described in the legend for Fig. S2. Download FIG S5, PDF file, 1.3 MB. Copyright © 2020 Nett et al. 2020 Nett et al. This content is distributed under the terms of the Creative Commons Attribution 4.0 International license.

The ability of different ancestral reconstructions to capture the likely vertical evolution of a gene cluster can be assessed by the number of events (loss, gain, and duplication) calculated by this method, with a lower number of events indicating a more parsimonious reconstruction. From this analysis, it was found that fewer evolutionary events are reconstructed in FO (75 events) than in FS (121 events) (c.f. Fig. S2 and S3), with the same relative trend observed with the partial trees (62 events for PO versus 78 events for PS) (c.f. Fig. S4 and S5). The greater parsimony (i.e., fewer reconstructed events) observed in reconstructions built with alignments of the concatenated GA operon strongly supports the previously suggested hypothesis of HGT among α-rhizobia (17). Accordingly, the reconstructions based on GA operon similarity (i.e., FO and PO) were used for further analyses of operon inheritance.

In contrast to the phytopathogens, a full-length *cyp115* gene is absent from the genomes of most rhizobia (including both α- and β-rhizobia). Instead, α- and β-rhizobia typically have only the core operon and, hence, can only produce the penultimate intermediate GA_9_ rather than bioactive GA_4_ (18, 20, 22). ROAGUE analysis indicates that *cyp115* loss occurred soon after α-rhizobial acquisition of the GA operon, as the reconstructed ancestral node that connects the α-rhizobia to *X. oryzae* (and the rest of the *Gammaproteobacteria*) does not contain *cyp115* (Fig. S5). Although the α-rhizobia presumably acquired their GA operon from a gammaproteobacterial ancestor, the *Gammaproteobacteria* seem to always have *cyp115* at the 5′ end of the operon. In contrast, the α-rhizobia typically only have a partial *cyp115* pseudogene/fragment located at this position, as previously described (14, 22). This suggests that the original operon acquired by an α-rhizobial ancestor contained *cyp115* and that this gene was subsequently lost. Interestingly, it was previously reported that *cyp115* is also absent from the GA operons of β-rhizobia, which seem to have independently gained their operon from a gammaproteobacterial progenitor (21).

Although *cyp115* is absent in most α-rhizobia, a subset of α-rhizobia (<20%) with the GA operon also have a full-length functional *cyp115*. However, only in one strain (*Mesorhizobium* sp. AA22) does the GA operon have *cyp115* in the same location as in gammaproteobacterial GA operons (22). Strikingly, ROAGUE analysis indicates that full-length *cyp115* has been regained independently in at least three different lineages, which is apparent in either the PS or PO reconstructions (Fig. S4 and S5). Indeed, other than in *Mesorhizobium* sp. AA22, these full-length *cyp115* genes reside in alternative locations relative to the rest of the GA operon (e.g., 3′ end of operon or distally located), as previously described (22), which further supports independent acquisition of *cyp115* via an additional HGT event.

Similar to that for *cyp115*, a full-length *idi* gene is generally present in the GA operons of *Gammaproteobacteria* and is only sporadically present in the GA operons of α-rhizobia. Collectively, 62 of the 118 α-rhizobial strains analyzed here possess this gene, which seems to invariably exhibit analogous positioning, i.e., at the 3′ end of the operon, as found in the gammaproteobacterial GA operons. Our ROAGUE analysis indicates that the ancestral strain with the GA operon likely possessed *idi* and that this gene has subsequently been lost in many α-rhizobial strains. However, there are notable differences among losses of this gene within the major α-rhizobial genera. For example, while the presence of *idi* appears to be stochastic within *Rhizobium* (16/26 strains) and *Sinorhizobium*/*Ensifer* (8/14), it is nearly absent from all *Bradyrhizobium* (2/40) but ubiquitously found in *Mesorhizobium* (36/36).

Not surprisingly, *ggps2* seems to be invariably associated with operons in which the canonical *ggps* is inactive (Fig. S4 and S5) and is only found in 13 of the 118 α-rhizobia analyzed in this study. However, the ancestral reconstructions further indicate that *ggps2* is present in two distinct clades in all trees; one composed of closely related *Rhizobium* strains and another with two *Bradyrhizobium* strains. While the *Rhizobium* all have homologous mutations in *ggps*, with similar positioning of *ggps2* (within 500 bp of the 3′ end of the operon), the two *Bradyrhizobium* strains have distinct *ggps* mutations, with *ggps2* positioned on opposite sides of the operon. There is higher homology between the GGPS2 proteins within each of the two clades than between them (Table S1), suggesting that each acquired *ggps2* independently. However, the lack of synteny between the two *Bradyrhizobium* strains, as well as distinct lesions in *ggps*, suggests that these each may have separately acquired *ggps2* as well. Given that the GGPS2 proteins are all much more closely related to each other (>83% amino acid sequence identity) than to any other homologs (<45% identity), *ggps2* appears to have undergone HGT within the α-rhizobia following initial acquisition by a GA operon in which *ggps* was lost/inactivated. Nevertheless, acquisition of *ggps2* was clearly followed by vertical transmission of the modified GA operon in the case of the larger and more homologous *Rhizobium*-containing clade.

In addition to ancestral gene loss and gain events, there also have been fusions between neighboring biosynthetic genes within the GA operon. In some α-rhizobia, the *fd*_GA_ gene, which is usually a distinct coding sequence, is found in-frame with either the 5′ proximal *cyp114* gene or the 3′ proximal *sdr*_GA_ gene. This results in *cyp114*-*fd* or *fd-sdr* fusions, respectively, which presumably encode bifunctional proteins. As fusion events are not analyzed by ROAGUE, these were assessed and categorized manually (see Table S4). The *cyp114-fd* fusion is only found in a single clade consisting almost entirely of *Rhizobium* species, which is most evident in the FO reconstruction (Fig. S3). In contrast, while the *fd-sdr* fusion is largely found in a clade consisting of predominantly *Mesorhizobium* species (Fig. S3), a similar fusion appears to have independently occurred in another clade as well. This fusion seems to be functional, as the activity of the fused Fd-SDR enzyme in M. loti MAFF303099 has been biochemically verified (4). Beyond the multiple observations of fused proteins in α-rhizobia, it should be noted that a functional *fd-sdr* fusion appears to have independently arisen in the β-rhizobia as well (21), further indicating that this is likely to not be functionally problematic. While functionality of the CYP114-Fd fusion has not been demonstrated to date, the cooperative activity of the two encoded enzymes (3) supports their ability to be functionally incorporated into a single polypeptide (33).

10.1128/mSphere.00292-20.9 TABLE S4 (a) List of strains containing a CYP114-Fd_GA_ gene fusion. (b) List of strains containing a Fd_GA_-SDR_GA_ gene fusion. Download Table S4, DOCX file, 0.1 MB. Copyright © 2020 Nett et al. 2020 Nett et al. This content is distributed under the terms of the Creative Commons Attribution 4.0 International license.

## DISCUSSION

**FIG 5:**
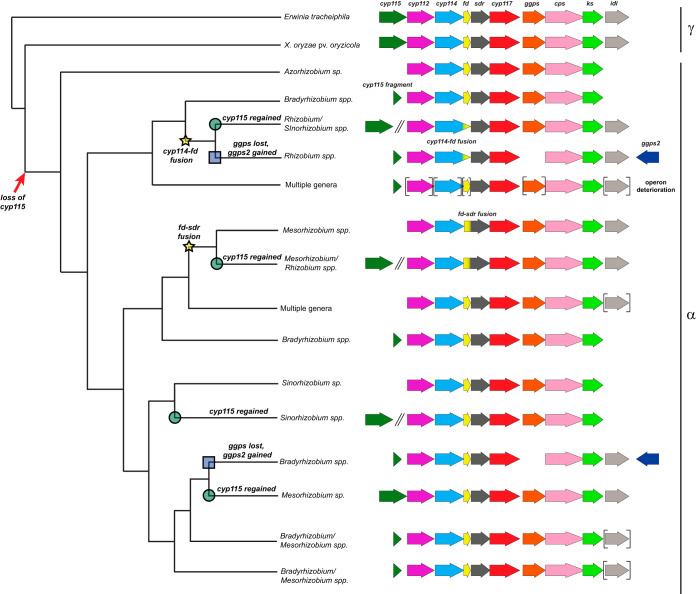
Summary of ancestral reconstruction for the GA biosynthetic operon. As a representation of GA operon evolution, the results from the full reconstruction generated using the concatenated operon (FO) are summarized here. Note that this figure is meant as a representation only; please see Fig. S3 in the supplemental material for the underlying data. Initial loss of the *cyp115* gene is indicated with a red arrow, while reacquisition of this gene is indicated with green circles at the ancestral nodes. Loss of *ggps* and acquisition of *ggps2* are indicated by blue boxes at the ancestral nodes. Gene fusion events (*cyp114-fd* and *fd-sdr*) are indicated with yellow stars. Brackets around a gene represent variable presence within that lineage. Double slanted lines indicate genes that are not located within the cluster (i.e., >500 bp away). The family of proteobacterial lineages is indicated to the right of the figure. α, *Alphaproteobacteria*; γ, *Gammaproteobacteria*.

Collectively, our analyses demonstrate a complex history of GA operon function, distribution, and evolution within the *Proteobacteria* (summarized in Fig. 5). Critical to this analysis was characterization of the *ggps2* and *idi* genes. Although these were previously noted to be associated with the GA operon, their function had not yet been demonstrated. To our knowledge, the *ggps2* and *idi* genes were the only remaining uncharacterized genes associated with the GA operon; thus, characterizing the enzymes encoded by these genes represents the final step in the elucidation of the associated biosynthetic capacity for the GA operon. Given that bacteria typically produce both isoprenoid precursors IPP and DMAPP directly via the methyl-erythritol-phosphate (MEP) pathway (23), an IDI is not strictly required, though it is possible that the presence of the *idi* gene would allow for increased flux toward GA by balancing precursor supply. Since the *idi* gene is ubiquitous in phytopathogen GA operons and has been lost multiple times in rhizobia, it may be that this gene optimizes GA production, which presumably serves to increase the virulence of the phytopathogens (9). However, the utility of optimized GA production by rhizobia is not evident. Thus, it is not clear why some α-rhizobia lineages retain this gene while others have lost it.

Unlike the isoprenoid precursor molecules, GGPP is not produced by many bacteria; thus, verification of *ggps2* as a GGPP synthase clarifies that GA biosynthesis is still possible in rhizobia where the original operon *ggps* is no longer functional. Interestingly, the more expansive *ggps2*-containing *Rhizobium* lineage also harbors a previously defined mutation in the *cps* gene that has been shown to affect product outcome (34). In particular, the otherwise conserved asparagine from the catalytic base dyad is replaced with a serine in this lineage, which results in predominant production of a distinct compound unrelated to GA biosynthesis (8β-hydroxy-*ent*-copalyl diphosphate) along with small amounts of the relevant GA intermediate (*ent*-copalyl diphosphate). Although the repeated vertical transmission of this modified operon suggests that, despite the presumably reduced flux, the associated production of GA still provides a selective advantage to these *Rhizobium* strains, it is tempting to speculate that this observation reflects genetic drift of the *cps* in the interlude between the loss of *ggps* and the acquisition of *ggps2*.

Both the GC content and ROAGUE analyses reported here are consistent with the hypothesis that the GA operon has undergone HGT between various plant-associated bacteria, including phytopathogenic gammaproteobacteria and symbiotic nitrogen-fixing α- and β-rhizobia. As an added layer of complexity, it is generally accepted that the large symbiotic or pathogenic genomic islands or plasmids (i.e., symbiotic or pathogenic modules), which enable the plant-associated lifestyle of these bacteria, are capable of undergoing HGT (35). For α-rhizobial strains where sufficient genomic information is available, the GA operon is invariably found within the symbiotic module (24, 28, 29, 36–38). Interestingly, HGT of the GA operon independently of the symbiotic module was previously suggested based on phylogenetic incongruences between genes representative of species (16S rRNA), symbiotic modules (*nifK*), and GA operon (*cps*) similarity (17). Thus, there appears to be multiple levels of HGT with the GA operon in α-rhizobia: (i) acquisition of the symbiotic module (i.e., symbiotic plasmid or genomic island), either with or without the GA operon, (ii) separate acquisition of the GA operon within the symbiotic module, and (iii) subsequent acquisition of auxiliary genes, including *ggps2* and *cyp115*.

Although widespread within proteobacteria, the GA operon has thus far only been found in plant-associated species (19). While this is not surprising due to the function of GA as a phytohormone, it emphasizes that such manipulation of host plants is an effective mechanism for bacteria to gain a selective advantage. Indeed, the ability to produce GA seems to be a powerful method of host manipulation for plant-associated microbes more generally, as certain phytopathogenic fungi also have convergently evolved the ability to produce GA as a virulence factor (8, 39).

Despite wide-ranging HGT of the GA operon between disparate classes of *Proteobacteria*, its scattered distribution within each of these classes strongly indicates that the ability to produce GA only provides a selective advantage under certain conditions. This is evident for both symbiotic rhizobia and bacterial phytopathogens. For example, the GA operon is selectively found in the *oryzicola* pathovar of *X. oryzae* (5), where the resulting GA acts as a virulence factor suppressing the plant jasmonic acid (JA)-induced defense response (9, 40, 41). In contrast, production of GA by M. loti MAFF303099 in symbiosis with Lotus japonicus limits the formation of additional nodules, apparently without a negative impact on plant growth (4).

The occurrence of GA operon fragments (i.e., presence of some, but not all, necessary biosynthetic operon genes) in many rhizobia indicates that the production of GA is not advantageous in all rhizobium-legume symbioses. For example, at the onset of this study, we identified 166 α-rhizobia with an obvious homolog of at least one GA operon gene, yet only 118 of these contained a gene cluster (i.e., two or more biosynthetic genes clustered together), and ∼20% of these clusters (26 of the 118 α-rhizobial operons analyzed here) are clearly nonfunctional due to the absence of key biosynthetic genes, consistent with dynamic selective pressure. It has been suggested that the GA operon is associated with species that inhabit determinate nodules (17), as these nodules grow via cell expansion (an activity commonly associated with GA signaling [42]), rather than indeterminate nodules, which grow via continuous cell division (43). However, while the presence of the GA operon does seem to be somewhat enriched within rhizobia that associate with determinate nodule-forming legumes (see Table S3 in the supplemental material), there are many examples of rhizobia with complete GA operons that were isolated from indeterminate nodules. For example, while most GA operon-containing *Bradyrhizobium* species associate with determinate nodule-forming plants, many species from the *Ensifer*/*Sinorhizobium*, *Mesorhizobium*, and *Rhizobium* genera with the operon were isolated from indeterminate nodules, as were several of the β-rhizobia with the GA operon (Integrated Microbe Genomes, JGI). Additionally, a number of rhizobia, including some with the GA operon such as *S. fredii* NGR234 (44), are capable of symbiosis with either type of legume, i.e., those forming either determinant or indeterminate nodules. These inconsistencies raise the question of why only some rhizobia have acquired and maintained the GA operon and, thus, the capacity to produce GA.

In addition to its scattered distribution, the operon exhibits notable genetic diversity within the α-rhizobia. For example, ROAGUE analysis indicates that loss of the usual *ggps* and subsequent recruitment of *ggps2* has been followed by HGT of this to other operons in which *ggps* has been inactivated. While it is difficult to infer the original source of *ggps2*, it is of note that the closest non-GA operon homologs are found clustered together with genes related to photosynthesis (e.g., in the alphaproteobacteria *Hyphomicrobium* sp. strain ghe19). Though this specific gene has not been functionally characterized, it seems reasonable that these homologs may be involved in producing GGPP as a precursor of photosynthetic pigments, i.e., phytol and/or carotenoids (26).

While the loss/gain of GGPP synthase genes represents rather dramatic events in GA operon evolution, other modifications to the operon may have subtle yet informative effects upon GA production. In particular, although loss of *idi* (and potentially the observed gene fusions events) may reduce the rate of GA production, it appears that this can be easily accommodated in certain rhizobium-legume pairings. Indeed, the expression of the GA operon is delayed in this symbiotic relationship (18), perhaps to mitigate any deleterious effects of GA during early nodule formation, which has been shown to be inhibitory to nodule formation, at least at higher concentrations (45).

Perhaps the most striking evolutionary aspect of the rhizobial GA operons is the early loss and scattered reacquisition of *cyp115* in α-rhizobia. While almost all α-rhizobial GA operons contain only remnants of *cyp115* at the position in the GA operon where it is found in gammaproteobacterial phytopathogens (22), there is one strain (*Mesorhizobium* sp. AA22) where a full-length copy is found at this location. Phylogenetic analysis further suggests that this *cyp115* from *Mesorhizobium* sp. AA22 is closest to the ancestor of all the full-length copies found in α-rhizobia, which are otherwise found at varied locations relative to the GA operon (22). The ROAGUE analysis reported here indicates that *cyp115* was lost shortly after acquisition of the ancestral GA operon by α-rhizobia, despite full-length copies being present in several different lineages. Accordingly, these results support the hypothesis that *cyp115* has been reacquired by this subset of rhizobia via independent HGT events. Notably, while not recognized in the original report (4), this includes M. loti MAFF303099, the only strain in which the biological role of rhizobial production of GA has been examined. Because *cyp115* is likely required for endogenous bacterial production of bioactive GA_4_ from the penultimate (inactive) precursor GA_9_, this highlights the question of the selective pressures driving evolution of GA biosynthesis in rhizobia.

The contrast between GA operon-containing bacterial lineages provides a captivating rationale for the further scattered distribution of *cyp115* in rhizobia. In particular, the GA operon in phytopathogens always contains *cyp115*, and these are thus capable of direct production of bioactive GA_4_, which serves to suppress the JA-induced plant defense response (9). This observation naturally leads to the hypothesis that rhizobial production of GA_4_ might negatively impact the ability of the host plant to defend against microbial pathogens invading the roots or root nodules, which would compromise the efficacy of this symbiotic interaction. Such detrimental effects from rhizobial production of bioactive GA_4_ may have driven the loss of *cyp115*. However, this would also result in a loss of GA signaling, as GA_9_, the product of an operon missing *cyp115*, presumably does not exert hormonal activity (46). One possible mechanism to compensate for *cyp115* loss would be legume host expression of the functionally equivalent plant GA 3-oxidase (GA3ox) gene (from endogenous plant GA metabolism) within the nodules in which the rhizobia reside. Expression of this plant gene would alleviate the necessity for rhizobial symbiont maintenance of *cyp115* and would further allow the host to control the production of bioactive GA_4_, thereby retaining the ability to mount an effective defense response when necessary. Reacquisition of *cyp115* might then be driven by a lack of such GA3ox expression in nodules by certain legumes. However, this scenario remains hypothetical; though precisely controlled GA production by the plant has been shown to be critical for normal nodulation to occur (45, 47), coordinated biosynthesis of GA_4_ by rhizobia and the legume host remains to be demonstrated. This would require both the transport of GA_9_ from the microbe to the host plant and the subsequent conversion of this precursor to a bioactive GA (e.g., GA_4_). Accordingly, continued study of the GA operon will provide insight into the various roles played by bacterium-produced GA in both symbiotic rhizobium-legume relationships and antagonistic plant-pathogen interactions, which in turn can be expected to provide fundamental knowledge regarding the ever-expanding roles of GA signaling in plants.

## MATERIALS AND METHODS

### Definition of α- and β-rhizobia abbreviations.

Within the manuscript, the abbreviated term α-rhizobia is used to refer to symbiotic nitrogen-fixing rhizobia that belong to the *Alphaproteobacteria* class. Likewise, the term β-rhizobia refers to symbiotic nitrogen-fixing rhizobia from the *Betaproteobacteria* class. For the α-rhizobia, the following genera were assessed because they have been found to contain the GA operon: *Azorhizobium*, *Bradyrhizobium*, *Ensifer/Sinorhizobium*, *Mesorhizobium*, *Microvirga*, and *Rhizobium* (20). The β-rhizobia previously found to have the GA operon fall within the *Burkholderia* and *Paraburkholderia* genera (21).

### Biochemical characterization of *Re*IDS2 and *Et*IDI.

*Re*IDS2 and *Et*IDI were cloned by amplification from genomic DNA of Rhizobium etli CE3, a streptomycin-resistant derivative of *R. etli* CFN 42 (48), and Erwinia tracheiphila PSU-1, respectively. PCR was performed with Q5 Hot Start High-Fidelity DNA polymerase (NEB) according to the product manual using gene-specific primers (see Table S5 in the supplemental material) and 5 μl of the high-GC-content enhancer for *Re*IDS2. The forward primers featured a 5′ CACC sequence to allow for directional cloning into pET101/D-TOPO (Invitrogen), as per the manufacturer’s instructions, such that each gene protein product would contain a C-terminal 6×His tag for purification. All subsequent routine cloning was performed using E. coli TOP10 cells. Each resulting plasmid construct was verified with Sanger sequencing to confirm successful ligation of the appropriate insert into the vector.

10.1128/mSphere.00292-20.10 TABLE S5 List of primers used in this study. Download Table S5, DOCX file, 0.1 MB. Copyright © 2020 Nett et al. 2020 Nett et al. This content is distributed under the terms of the Creative Commons Attribution 4.0 International license.

For recombinant expression, pET101 constructs containing either *Re*IDS2 or *Et*IDI were transformed into E. coli strain BL21 Star (Invitrogen). Starter cultures were inoculated in 10 ml NZY medium (10 g liter^−1^ NaCl, 10 g liter^−1^ casein, 5 g liter^−1^ yeast extract, 1 g liter^−1^ anhydrous MgSO_4_, pH 7.0) with 50 μg ml^−1^ carbenicillin and grown at 18°C with 200-rpm shaking for 3 days. A portion (5 ml) of these starter cultures was used to inoculate 100 ml fresh NZY medium containing 50 μg ml^−1^ carbenicillin, which were grown at 18°C with 200 rpm. After reaching an optical density at 600 nm (OD_600_) of 0.6, the protein production was induced with 1 mM isopropyl-β-d-thiogalactopyranoside (IPTG) and grown under continuous shaking at 200 rpm at 18°C for 24 h. Cells were harvested by centrifugation at 5,000 × *g* for 15 min. The cell pellet was resuspended in 5 ml 3-(*N*-morpholino)-2-hydroxypropanesulfonic acid (MOPSO) buffer (25 mM MOPSO [pH 7.2], 10 mM MgCl_2_, 10% glycerol) with 20 mM imidazole and then lysed using an EmulsiFlex C-5 homogenizer (Avestin, Canada). The homogenized suspensions were centrifuged at 16,000 × *g* for 60 min to pellet cell debris. The resulting supernatant was passed over 1 ml Ni-nitrilotriacetic acid (NTA) agarose (Qiagen), which was then washed with 5 ml buffer containing 20 mM imidazole and then with an additional 5 ml of buffer with 50 mM imidazole. The recombinant 6×His-tagged proteins were eluted with 2 ml buffer containing 250 mM imidazole.

*Re*IDS2 enzyme assays were carried out in triplicates with 10 μg of purified heterologously expressed protein in 300 μl of buffer and 50 μM IPP and 50 μM DMAPP as the substrates. *Et*IDI assays were also performed in triplicates using a combined assay of 20 μg *Re*IDS2 (following confirmation as a GGPP synthase, renamed *Re*GGPS2) and 20 μg of *Et*IDI, with the addition of 10 μM flavin mononucleotide and 5 mM NADPH as described previously (49) and either 100 μM IPP or DMAPP. Assays were incubated for 2 h at 30°C, flash frozen in liquid nitrogen, and kept at −80°C until their analysis by liquid chromatography-tandem mass spectrometry (LC-MS/MS), which was carried out as previously described (50).

### Analysis of GA operon GC content.

GA operons for representative strains of alpha- and gammaproteobacteria were identified via BLAST searches against the NCBI nonredundant nucleotide database (https://blast.ncbi.nlm.nih.gov/Blast.cgi) by using individual GA operon gene sequences as the query. Complete GA operon sequences along with ∼10 kb of genomic sequence flanking the operon on both sides were downloaded from NCBI. Sequences were analyzed in Geneious Prime (Biomatters, Ltd.), where GC content was determined for a sliding window size of 500 bp.

### Operon phylogenetic reconstruction.

**(i) General methodology and source code.** A summary of the methods used herein can be found in Fig. S1. All code and scripts used for analysis within the manuscript, as well as a general workflow for the use of the ROAGUE method in the ancestral reconstruction of gene blocks, can be found at the following GitHub repositories: https://github.com/nguyenngochuy91/Gibberellin-Operon and https://github.com/nguyenngochuy91/Ancestral-Blocks-Reconstruction.

**(ii) Identification of orthologous gene blocks.** The terms reference taxa, neighboring genes, gene blocks, events, and orthologous gene blocks (orthoblocks) were described previously (25). Briefly, the reference taxon is a strain in which the operon in question has been experimentally validated. Two genes are considered neighboring genes if they are 500 nucleotides or fewer apart and on the same strand. A gene block comprises no fewer than two such neighboring open reading frames. Organisms have orthoblocks when each has at least two neighboring genes that are homologous to genes in a gene block in the reference taxon’s genome. An event is a change in the gene block between any two species with homologous gene blocks. We identified three types of pairwise events between orthoblocks in different taxa: splits, deletions, and duplications. The event-based distance between any two orthoblocks is the sum of the minimized count of splits, duplications, and deletions.

Using Xanthomonas oryzae pv. *oryzicola* BLS256 (Xoc) as a reference taxon, we retrieved the 10 genes in the GA operon (*cyp115*, *cyp112*, *cyp114*, *fd*_GA_, *sdr*_GA_, *cyp117*, *ggps*, *cps*, *ks*, and *idi*), for which all genes, or their orthologs (with the exception of *idi*), were previously experimentally validated (5, 9, 17). From those 10 genes, we determined whether a query strain contains orthologous gene blocks via BLAST searches against the NCBI nonredundant nucleotide database. Sequences were confirmed as orthologs if the BLAST E value was 10^−10^ or less. The initial BLAST analysis (12 April 2017) revealed 166 bacterial strains within the *Alphaproteobacteria* class (specifically, *Azorhizobium*, *Bradyrhizobium*, *Mesorhizobium*, *Microvirga*, *Rhizobium*, and *Ensifer*/*Sinorhizobium* species) that contained orthologs of one or more of the GA operon genes. Given a set of 166 species/strain names, the corresponding genome assembly files were retrieved from the NCBI website. Using their assembly_summary.txt file, the strains’ genomic fna (fasta nucleic acid) files were downloaded. The number of strains analyzed was further reduced by only including strains with multiple GA operon genes (>2) clustered together, resulting in a final total of 118 strains (listed in Table S3). Retrieved genome assemblies for these strains were then annotated using Prokka (51).

**(iii) Identification of pseudo-*cyp115* sequences.** Though *cyp115* is found as a full-length gene in the majority of gammaproteobacterial GA operons, as well as in some alphaproteobacterial operons, it exists as a truncated open reading frame, or gene fragment, in most *Alphaproteobacteria*. Previous assessment of *cyp115* gene fragments was performed through manual assessment of the genomic sequence 5′ to *cyp112* in the GA operon (22). To identify these gene fragments (pseudo-*cyp115*, or p-*cyp115*) in a more streamlined process, BLAST searches were performed with the Xoc *cyp115*, as described above, to identify sequences with a BLAST E value of less than 10^−10^. If the length of the identified sequence was less than 60% that of the query gene and shared greater than 50% sequence identity, then the sequence was annotated as p-*cyp115*.

**(iv) Computational reconstruction of the gibberellin operon phylogeny.** ROAGUE (Reconstruction of Ancestral Gene blocks Using Events) software was used to reconstruct ancestral gene blocks. ROAGUE accepts as input (i) a set of extant bacterial genomes, (ii) a phylogenetic tree describing the relatedness between the set of species, and (iii) a gold standard operon that has been experimentally validated from one species in the set of given genomes. ROAGUE finds the orthologs of the genes in the reference operons and then constructs the hypothesized ancestral gene blocks using a maximum parsimony algorithm, as previously described (31).

A previous phylogenetic analysis demonstrated incongruences between phylogenetic trees constructed with a species marker (16S rRNA), a symbiotic module marker (*nifK*), and GA operon genes (17). This suggests independent horizontal gene transfer (HGT) of both the symbiotic module and the GA operon. In an effort to objectively and thoroughly assess the possibility of HGT of the GA operon among alphaproteobacterial rhizobia, phylogenetic trees were constructed using alignments from both a species marker gene (*rpoB*) and from a concatenation of the protein sequences for genes in the GA operon. The species tree (S) was generated by aligning *rpoB* (species marker) protein sequences via the MUSCLE algorithm (https://www.ebi.ac.uk/Tools/msa/muscle/) and then using the neighbor-joining method to generate the tree. For the operon tree (O), the protein sequences of open reading frames (ORFs) of the orthoblock genes for each species were naively concatenated. Because many species lack full-length *cyp115* and *idi* genes, and due to the loss of *ggps* in several species, only the following genes, which seem to be more uniformly conserved, were concatenated for this purpose: *cyp112*, *cyp114*, *fd*_GA_, *sdr*_GA_, *cyp117*, *cps*, and *ks*. A multiple-sequence alignment of these concatenations was performed using the MUSCLE algorithm, and the neighbor-joining method was used to build the presented trees. Erwinia tracheiphila PSU-1 was used as the phylogenetic outgroup in both trees, as this is distant phylogenetically and has been shown to have the most distant GA operon to that of the *Alphaproteobacteria* (20). ROAGUE analysis (31) was then applied to each phylogenetic reconstruction. Each leaf node *v* in S and O contains orthologs to the genes found in the GA operon of the reference species (Xoc). For any two genes *a* and *b*, if the chromosomal distance was less than 500 bp, the genes were written as *ab*. If the distance was greater than 500 bp, they were written with the separator character as *a*|*b*.

Due to the large number (118 strains) and redundancy (both phylogenetically and in operon structure) of the alphaproteobacterial strains, the size of the tree was reduced by using the Phylogenetic Diversity Analyzer software (32). To facilitate analysis and presentation, the number of species was reduced to 64, which was still representative of the overall diversity and enables ready visualization. Additionally, this software only keeps species that have a sufficiently unique sequence identity to give distinct branches on the phylogenetic tree (i.e., reflecting appreciable distance between species). Accordingly, this approach also eliminated the redundancy that would otherwise have confounded this analysis. The full operon tree, full species tree, partial operon tree, and partial species tree are referred to as FO, FS, PO, and PS trees, respectively. The topology for each of these reconstructions was then compared in order to identify major incongruences that may indicate HGT.

**(iv) Identification of gene fusion events.** Currently, ROAGUE does not account for gene fusion. Given the presence of several GA operons wherein the *fd*_GA_ gene is fused in frame with either the *sdr*_GA_ gene or the *cyp114* gene, it was necessary to assess these manually. This was accomplished by reassessing the gene block to determine if the *fd*_GA_ gene was missing, as the fusion of this gene in-frame with either *sdr*_GA_ or *cyp114* would result in it not being recognized as a unique ORF by the ROAGUE software. Then, the initial BLAST results with *fdGA* as query were analyzed and potential fusions were found by checking the start and end of the alignments of the subject genes (i.e., *cyp114* and *sdrGA*). Strains with *cyp114*-*fd* fusions are listed in Table S4a, and strains with *fd*-*sdr* fusions are listed in Table S4b.
