# Supplementary material for: Unraveling a Tangled Skein: Evolutionary Analysis of the Bacterial Gibberellin Biosynthetic Operon
Source: mSphere. 2020 Jun 3;5(3):e00292-20. doi: 10.1128/mSphere.00292-20 (PMC7273348; doi:10.1128/mSphere.00292-20)
Supplement: TABLE S1 [file mSphere.00292-20-st001.docx]

**Supplemental Table 1. Pairwise alignments of GGPS2 proteins and representative GGPS proteins.** Protein sequences were aligned using the MUSCLE algorithm (Geneious Prime; default settings). The GGPS proteins included in this analysis were selected due to the relatively close phylogenetic relationship between the core operon of this strain and that of the *ggps2*-containing strains, which lack a full-length *ggps* gene (i.e. *Rhizobium* *etli* bv. *mimosae* str. Mim1 has the most similar core operon to the *ggps2*-containing *Rhizobium* strains and *Bradyrhizobium* sp. WSM1417 has the most similar core operon to *ggps2*-containing *Bradyrhizobium* strains). Sequences with >90% amino acid identity are highlighted in dark blue, while those with >80% but less than 90% amino acid identity are highlighted in light blue.
