# Supplementary material for: Unraveling a Tangled Skein: Evolutionary Analysis of the Bacterial Gibberellin Biosynthetic Operon
Source: mSphere. 2020 Jun 3;5(3):e00292-20. doi: 10.1128/mSphere.00292-20 (PMC7273348; doi:10.1128/mSphere.00292-20)
Supplement: TABLE S2 [file mSphere.00292-20-st002.docx]

**Supplemental Table 2. Percent GC content in the core GA operon (*cyp112*-*ks*) compared to *ggps2*.** Shown are the results for representative *ggps2*-containing strains analyzed in this study. Also included is the difference in percent GC content (delta) between the core GA operon and *ggps2*.

|  | ***%GC*** | |  |
| --- | --- | --- | --- |
| **strain** | ***cyp112*-*ks*** | ***ggps2*** | **delta (%)** |
| *Bradyrhizobium sp.* WSM2254 | 68.8 | 62.9 | 5.9 |
| *Bradyrhizobium sp.* WSM3983 | 69.1 | 62.3 | 6.8 |
| *Rhizobium etli* CFN 42 | 66.0 | 59.1 | 6.9 |
| *Rhizobium* sp. CCGE 510 | 66.2 | 59 | 7.2 |
| *Rhizobium* sp. HBR26 | 66.1 | 59.4 | 6.7 |
