# Supplementary material for: Unraveling a Tangled Skein: Evolutionary Analysis of the Bacterial Gibberellin Biosynthetic Operon
Source: mSphere. 2020 Jun 3;5(3):e00292-20. doi: 10.1128/mSphere.00292-20 (PMC7273348; doi:10.1128/mSphere.00292-20)
Supplement: TABLE S3 [file mSphere.00292-20-st003.docx]

**Supplemental Table 3. List of alphaproteobacteria strains used within the ancestral reconstruction analyses.** Shown for each strain is the corresponding GenBank genome accession, the legume host from which it was isolated, and the nodule type (D = determinate, I = indeterminate, * = neither). Legume host of isolation were identified through the Joint Genomes Institue (JGI) Microbial Genomes and Microbiomes (IMG/M) database entry for each strain. Nodule type was confirmed through literature searches of each legume host.

| **GenBank genome accession** | **strain** | **legume host** | **nodule type** |
| --- | --- | --- | --- |
| AXBA01000001.1 | *Azorhizobium doebereinerae* UFLA1-100 | *Sesbania virgata* | * |
| AP014659.1 | *Bradyrhizobium diazoefficiens* NK6 | *Glycine max* | D |
| ADOU02000001.1 | *Bradyrhizobium diazoefficiens* SEMIA 5080 | *Glycine max* | D |
| BA000040.2 | *Bradyrhizobium diazoefficiens* USDA 110 | *Glycine max* | D |
| AXAH01000003.1 | *Bradyrhizobium elkanii* USDA 3254 | *Phaseolus acutifolius* | D |
| AXAW01000001.1 | *Bradyrhizobium elkanii* USDA 3259 | *Phaseolus lunatus* | D |
| KB900701.1 | *Bradyrhizobium elkanii* USDA 76 | *Glycine max* | D |
| AXAU01000001.1 | *Bradyrhizobium elkanii* WSM1741 | *Rhynchosia minima* | D |
| LFIP02000001.1 | *Bradyrhizobium embrapense* SEMIA 6208 | *Desmodium heterocarpon* | D |
| AXBC01000011.1 | *Bradyrhizobium* genosp. SA-4 str. CB756 | *Macrotyloma africanum* | D |
| CP010313.1 | *Bradyrhizobium japonicum* E109 | *Glycine max* | D |
| JGCL01000001.1 | *Bradyrhizobium japonicum* FN1 | *Glycine max* (soil isolate) | D |
| LGUJ01000001.1 | *Bradyrhizobium japonicum* Is-1 | *Glycine max* | D |
| JRPN01000001.1 | *Bradyrhizobium japonicum* Is-34 | *Glycine max* | D |
| CP007569.1 | *Bradyrhizobium japonicum* SEMIA 5079 | *Glycine max* | D |
| AXAX01000001.1 | *Bradyrhizobium japonicum* USDA 122 | *Glycine max* | D |
| AXVP01000001.1 | *Bradyrhizobium japonicum* USDA 123 | *Glycine max* | D |
| KB893805.1 | *Bradyrhizobium japonicum* USDA 124 | *Glycine max* | D |
| AXAT01000001.1 | *Bradyrhizobium japonicum* USDA 135 | *Glycine max* | D |
| AXAG01000003.1 | *Bradyrhizobium japonicum* USDA 38 | *Glycine max* | D |
| AXAF01000003.1 | *Bradyrhizobium japonicum* USDA 4 | *Glycine max* | D |
| AP012206.1 | *Bradyrhizobium japonicum* USDA 6 | *Glycine max* | D |
| LJYE01000001.1 | *Bradyrhizobium pachyrhizi* BR3262 | *Vigna unguiculata* | D |
| LFIQ01000001.1 | *Bradyrhizobium pachyrhizi* PAC 48 | *Pachyrhizus erosus* | D |
| AJQG01000001.1 | *Bradyrhizobium* sp. CCBAU 15615 | *Glycine max* | D |
| AJQH01000001.1 | *Bradyrhizobium* sp. CCBAU 15635 | *Glycine max* | D |
| AJQE01000001.1 | *Bradyrhizobium* sp. CCBAU 43298 | *Glycine max* | D |
| AUFA01000001.1 | *Bradyrhizobium* sp. Cp5.3 | *Centrosema pubescens* | D |
| FMAI01000001.1 | *Bradyrhizobium* sp. ERR11 | *Erythrina brucei* | D |
| AUGA01000001.1 | *Bradyrhizobium* sp. th.b2 | *Amphicarpaea bracteata* | D |
| AXAD01000001.1 | *Bradyrhizobium* sp. USDA 3384 | *Kennedia coccinea* | D |
| JH600072.1 | *Bradyrhizobium* sp. WSM1253 | *Ornithopus compressus* | D |
| KI911783.1 | *Bradyrhizobium* sp. WSM1417 | *Lupinus sp.* | I |
| AXAZ01000001.1 | *Bradyrhizobium* sp. WSM1743 | *Indigofera* sp. | I |
| AXAB01000001.1 | *Bradyrhizobium* sp. WSM2254 | *Acacia dealbata* | I |
| KB902768.1 | *Bradyrhizobium* sp. WSM2793 | *Rhynchosia totta* | D |
| AXAY01000001.1 | *Bradyrhizobium* sp. WSM3983 | *Kennedia coccinea* | D |
| KB890498.1 | *Bradyrhizobium* sp. WSM4349 | *Syrmatium glabrum (Lotus scoparius)* | D* |
| CM001442.1 | *Bradyrhizobium* sp. WSM471 | *Ornithopus pinnatus* | D |
| LJYF01000001.1 | *Bradyrhizobium yuanmingense* BR3267 | *Kennedia coccinea* | D |
| AJQL01000001.1 | *Bradyrhizobium yuanmingense* CCBAU 35157 | *Glycine max* | D |
| AJQT01000001.1 | *Ensifer sojae* CCBAU 5684 | *Glycine max* | D |
| AZNX01000001.1 | *Ensifer* sp. TW10 | *Tephrosia purpurea* | I |
| AZUW01000001.1 | *Ensifer* sp. WSM1721 | *Indigofera sp.* | I |
| AHAM01000001.1 | *Mesorhizobium alhagi* CCNWXJ12-2 | *Alhagi sparsifolia* | I |
| AGSN01000001.1 | *Mesorhizobium amorphae* CCNWGS0123 | *Robinia pseudoacacia* | I |
| CP003358.1 | *Mesorhizobium australicum* WSM2073 | *Biserrula pelecinus* | I |
| CP002447.1 | *Mesorhizobium ciceri* bv. *biserrulae* WSM1271 | *Biserrula pelecinus* | I |
| CP015064.1 | *Mesorhizobium ciceri* bv. *biserrulae* WSM1284 | *Biserrula pelecinus* | I |
| AXAE01000005.1 | *Mesorhizobium erdmanii* USDA 3471 | *Lotus corniculatus* | D |
| AXAL01000001.1 | *Mesorhizobium loti* CJ3sym | *Lotus corniculatus* | D |
| AP003017.1 | *Mesorhizobium loti* MAFF303099 | *Lotus pedunculatus* | D |
| LYTJ01000001.1 | *Mesorhizobium loti* NZP2014 | *Lotus sp.* | D |
| KB913026.1 | *Mesorhizobium loti* NZP2037 | *Lotus divaricatus* | D |
| LYTK01000001.1 | *Mesorhizobium loti* NZP2042 | *Lotus sp.* | D |
| KI632510.1 | *Mesorhizobium loti* R7A | *Lotus corniculatus* | D |
| KI912159.1 | *Mesorhizobium loti* R88b | *Lotus corniculatus* | D |
| CP002279.1 | *Mesorhizobium opportunistum* WSM2075 | *Biserrula pelecinus* | I |
| LYTO01000001.1 | *Mesorhizobium* sp. AA22 | *Biserrula pelecinus L* | I |
| AYXF01000001.1 | *Mesorhizobium* sp. L103C105A0 | *Acmispon wrangelianus* | D |
| AYWZ01000001.1 | *Mesorhizobium* sp. L2C066B000 | *Acmispon wrangelianus* | D |
| AYWW01000001.1 | *Mesorhizobium* sp. L2C085B000 | *Acmispon wrangelianus* | D |
| AYWU01000001.1 | *Mesorhizobium* sp. L48C026A00 | *Acmispon wrangelianus* | D |
| AYWP01000001.1 | *Mesorhizobium* sp. LNHC232B00 | *Acmispon wrangelianus* | D |
| AYWN01000001.1 | *Mesorhizobium* sp. LNJC372A00 | *Acmispon wrangelianus* | D |
| AYWB01000001.1 | *Mesorhizobium* sp. LSHC412B00 | *Acmispon wrangelianus* | D |
| AYWA01000001.1 | *Mesorhizobium* sp. LSHC414A00 | *Acmispon wrangelianus* | D |
| AYVY01000001.1 | *Mesorhizobium* sp. LSHC420B00 | *Acmispon wrangelianus* | D |
| AYVX01000001.1 | *Mesorhizobium* sp. LSHC422A00 | *Acmispon wrangelianus* | D |
| AYVS01000001.1 | *Mesorhizobium* sp. LSHC440B00 | *Acmispon wrangelianus* | D |
| AYVP01000001.1 | *Mesorhizobium* sp. LSJC265A00 | *Acmispon wrangelianus* | D |
| AYVO01000001.1 | *Mesorhizobium* sp. LSJC268A00 | *Acmispon wrangelianus* | D |
| AYVN01000001.1 | *Mesorhizobium* sp. LSJC269B00 | *Acmispon wrangelianus* | D |
| AYVM01000001.1 | *Mesorhizobium* sp. LSJC277A00 | *Acmispon wrangelianus* | D |
| MDLH01000001.1 | *Mesorhizobium* sp. SEMIA 3007 | *Pisum sativum* | I |
| CAAF010000001.1 | *Mesorhizobium* sp. STM 4661 | *Anthyllis vulneraria* | D |
| AZUV01000001.1 | *Mesorhizobium* sp. WSM1293 | *Lotus sp.* | D |
| AZUX01000001.1 | *Mesorhizobium* sp. WSM2561 | *Lessertia diffusa* | I |
| ATYO01000001.1 | *Mesorhizobium* sp. WSM3224 | *Otholobium candicans* | D |
| AZUY01000001.1 | *Mesorhizobium* sp. WSM3626 | *Lessertia diffusa* | I |
| AZYE01000987.1 | *Microvirga lupini* Lut6 | *Lupinus texensis* | I |
| LJSR01000001.1 | *Rhizobium acidisoli* FH23 | *Phaseolus vulgaris* | D |
| LFIO01000001.1 | *Rhizobium ecuadorense* CNPSO 671 | *Phaseolus vulgaris* | D |
| CP006986.1 | *Rhizobium etli* bv. *mimosae* str. IE4771 | *Phaseolus vulgaris* | D |
| CP005950.1 | *Rhizobium etli* bv. *mimosae* str. Mim1 | *Mimosa affinis* | I |
| CP007641.1 | *Rhizobium etli* bv. *phaseoli* str. IE4803 | *Phaseolus vulgaris* | D |
| CP000133.1 | *Rhizobium etli* CFN 42 | *Phaseolus vulgaris* | D |
| CP001074.1 | *Rhizobium etli* CIAT 652 | *Phaseolus vulgaris* | D |
| ATTO01000001.1 | *Rhizobium favelukesii* OR191 | *Medicago sativa* | I |
| AQHN01000001.1 | *Rhizobium freirei* PRF 81 | *Phaseolus vulgaris* | D |
| CP006877.1 | *Rhizobium gallicum* bv. *gallicum* R602 | *Phaseolus vulgaris* | D |
| AEYE02000001.1 | *Rhizobium grahamii* CCGE 502 | *Dalea leporin* | I |
| KB905373.1 | *Rhizobium leguminosarum* bv. *phaseoli* 4292 | *Phaseolus vulgaris* | D |
| JFGP01000001.1 | *Rhizobium leguminosarum* bv. *phaseoli* CCGM1 | *Phaseolus vulgaris* | D |
| ATTN01000001.1 | *Rhizobium leguminosarum* bv. *phaseoli* FA23 | *Phaseolus vulgaris* | D |
| AJUI01000023.1 | *Rhizobium leguminosarum* bv. *trifolii* CC278f | *Trifolium nanum* | I |
| KI911771.1 | *Rhizobium leguminosarum* bv. *trifolii* CC283b | *Trifolium ambiguum* | I |
| ATYQ01000001.1 | *Rhizobium leguminosarum* bv. *viciae* VF39 | *Vicia faba* | I |
| AUFB01000001.1 | *Rhizobium leucaenae* USDA 9039 | *Phaseolus vulgaris* | D |
| FMAF01000001.1 | *Rhizobium lusitanum* P1-7 | *Phaseolus vulgaris* | D |
| HF536772.1 | *Rhizobium mesoamericanum* STM3625 | *Mimosa pudica* | I |
| ATYY01000001.1 | *Rhizobium mesoamericanum* STM6155 | *Mimosa pudica* | I |
| ATTQ01000001.1 | *Rhizobium mongolense* USDA 1844 | *Medicago ruthenica* | I |
| AHJU02000001.1 | *Rhizobium phaseoli* Ch24-10 | *Phaseolus vulgaris* (maize stem isolate) | D |
| AEYF01000001.1 | *Rhizobium* sp. CCGE 510 | *Phaseolus albescens* | D |
| FMAJ01000001.1 | *Rhizobium* sp. HBR26 | *Phaseolus vulgaris* | D |
| CP004015.1 | *Rhizobium tropici* CIAT 899 | *Phaseolus vulgaris* | D |
| JFGO01000001.1 | *Sinorhizobium americanum* CCGM7 | *Phaseolus vulgaris* | D |
| ATYB01000007.1 | *Sinorhizobium arboris* LMG 14919 | *Prosopis chilensis* | I |
| AJQN01000001.1 | *Sinorhizobium fredii* CCBAU 25509 | *Glycine max* | D |
| AMCX01000001.1 | *Sinorhizobium fredii* GR64 | *Phaseolus vulgaris* | D |
| HE616890.1 | *Sinorhizobium fredii* HH103 | *Glycine max* (soil isolate) | D |
| CP000874.1 | *Sinorhizobium fredii* NGR234 | *Lablab purpureus* | D |
| CP003563.1 | *Sinorhizobium fredii* USDA 257 | *Glycine max* | D |
| AQWP01000001.1 | *Sinorhizobium meliloti* 4H41 | *Phaseolus vulgaris* | D |
| ATZC01000001.1 | *Sinorhizobium meliloti* GVPV12 | *Phaseolus vulgaris* | D |
| ATYC01000007.1 | *Sinorhizobium meliloti* WSM4191 | *Melilotus siculus* | I |
| LATE01000001.1 | *Sinorhizobium* sp. PC2 | *Prosopis cineraria* | I |
