# Supplementary figures and images for: Unraveling a Tangled Skein: Evolutionary Analysis of the Bacterial Gibberellin Biosynthetic Operon
Source: mSphere. 2020 Jun 3;5(3):e00292-20. doi: 10.1128/mSphere.00292-20 (PMC7273348; doi:10.1128/mSphere.00292-20)

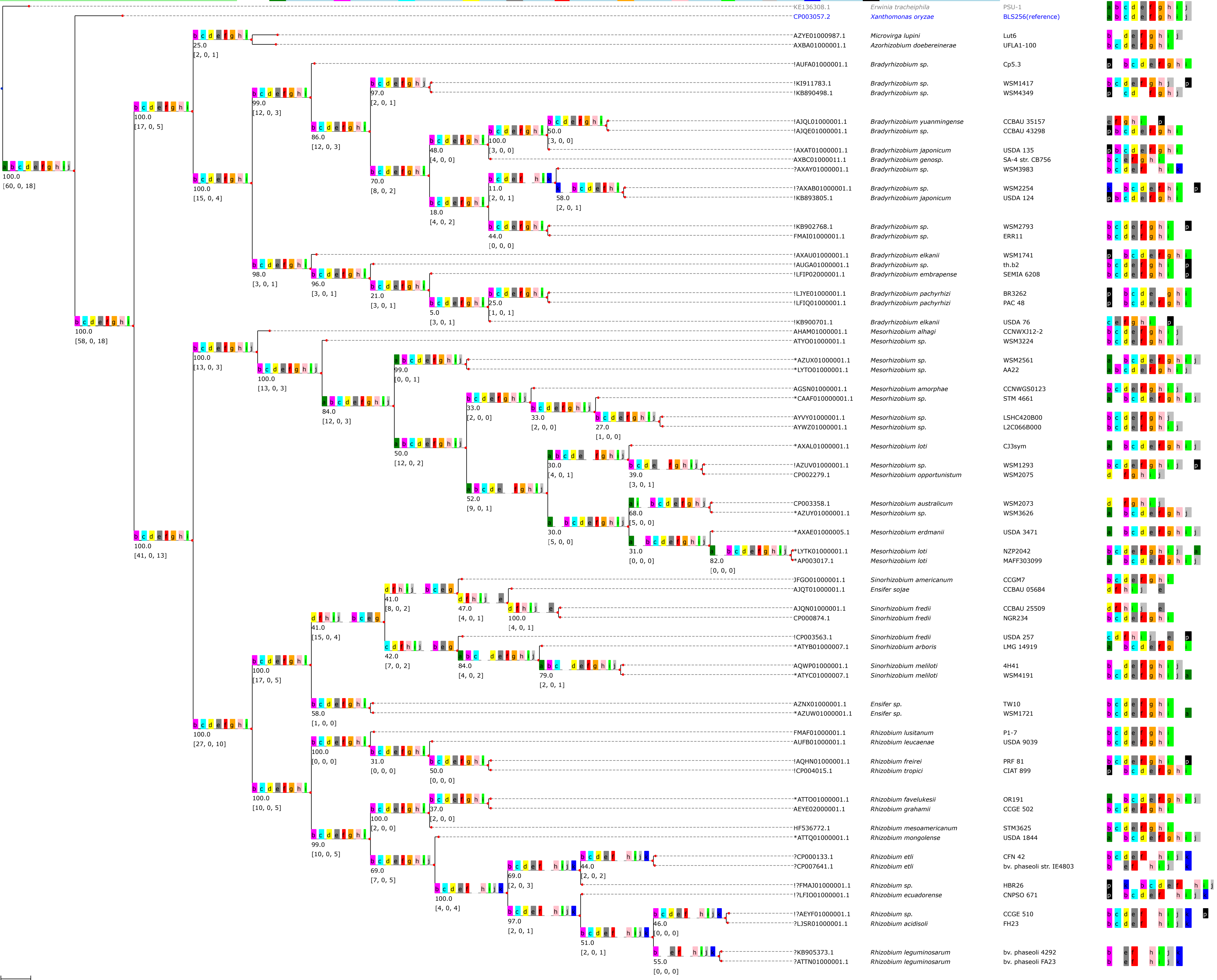

Supplement: FIG S4 [file mSphere.00292-20-sf004.pdf]

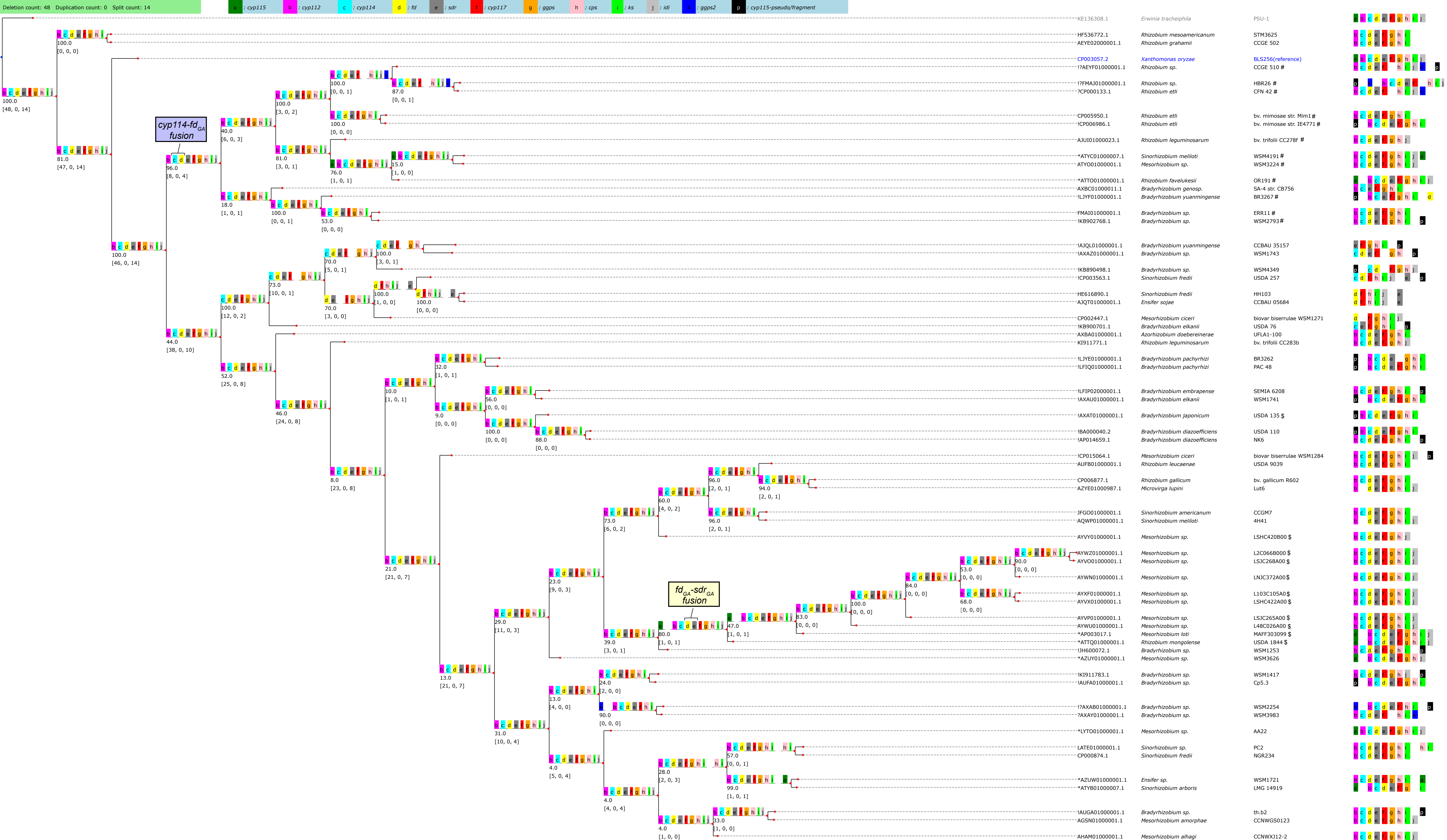

Supplement: FIG S5 [file mSphere.00292-20-sf005.pdf]
