# Supplementary material for: Unraveling a Tangled Skein: Evolutionary Analysis of the Bacterial Gibberellin Biosynthetic Operon
Source: mSphere. 2020 Jun 3;5(3):e00292-20. doi: 10.1128/mSphere.00292-20 (PMC7273348; doi:10.1128/mSphere.00292-20)
Supplement: TABLE S4 [file mSphere.00292-20-st004.docx]

**Supplemental Table 4a. List of strains containing a CYP114-Fd_GA_ gene fusion.**

| *Bradyrhizobium* sp. ERR11 |
| --- |
| *Bradyrhizobium yuanmingense* BR3267 |
| *Bradyrhizobium* sp. WSM2793 |
| *Mesorhizobium* sp. WSM3224 |
| *Rhizobium acidisoli* FH23 |
| *Rhizobium ecuadorense* CNSPO 671 |
| *Rhizobium etli* CIAT 652 |
| *Rhizobium etli* CFN 42 |
| *Rhizobium etli* bv. *mimosae* str. Mim1 |
| *Rhizobium etli* bv. *mimosa* str. IE4771 |
| *Rhizobium etli* bv. *phaseoli* IE4803 |
| *Rhizobium favelukesii* OR191 |
| *Rhizobium leguminosarum* bv. *phaseoli* 4292 |
| *Rhizobium leguminosarum* bv. *phaseoli* CCGM1 |
| *Rhizobium leguminosarum* bv. *phaseoli* FA23 |
| *Rhizobium leguminosarum* bv. *trifolii* CC278f |
| *Rhizobium phaseoli* Ch24-10 |
| *Rhizobium* sp. HBR26 |
| *Rhizobium* sp. CCGE 510 |
| *Sinorhizobium meliloti* WSM4191 |

**Supplemental Table 4b. List of strains containing a Fd_GA_-SDR_GA_ gene fusion.**

| *Bradyrhizobium japonicum* USDA 135 |
| --- |
| *Bradyrhizobium* sp. WSM1253 |
| *Bradyrhizobium* sp. WSM1417 |
| *Bradyrhizobium* sp. WSM471 |
| *Mesorhizobium loti* CJ3sym |
| *Mesorhizobium loti* MAFF303099 |
| *Mesorhizobium loti* NZP2014 |
| *Mesorhizobium loti* NZP2037 |
| *Mesorhizobium loti* NZP2042 |
| *Mesorhizobium loti* R7A |
| *Mesorhizobium loti* R88b |
| *Mesorhizobium* sp. L103C105A0 |
| *Mesorhizobium* sp. L2C066B000 |
| *Mesorhizobium* sp. L2C085B000 |
| *Mesorhizobium* sp. L48C026A00 |
| *Mesorhizobium* sp. LNHC232B00 |
| *Mesorhizobium* sp. LNJC372A00 |
| *Mesorhizobium* sp. LSHC414A00 |
| *Mesorhizobium* sp. LSHC412B00 |
| *Mesorhizobium* sp. LSHC420B00 |
| *Mesorhizobium* sp. LSHC422A00 |
| *Mesorhizobium* sp. LSHC440B00 |
| *Mesorhizobium* sp. LSJC265A00 |
| *Mesorhizobium* sp. LSJC268A00 |
| *Mesorhizobium* sp. LSJC269B00 |
| *Mesorhizobium* sp. LSJC277A00 |
| *Mesorhizobium* sp. SEMIA 3007 |
| *Mesorhizobium* sp. STM 4661 |
| *Rhizobium mongolense* USDA 1844 |
