# Supplementary material for: Unraveling a Tangled Skein: Evolutionary Analysis of the Bacterial Gibberellin Biosynthetic Operon
Source: mSphere. 2020 Jun 3;5(3):e00292-20. doi: 10.1128/mSphere.00292-20 (PMC7273348; doi:10.1128/mSphere.00292-20)
Supplement: TABLE S5 [file mSphere.00292-20-st005.docx]

**Supplemental Table 5.** **List of primers used in this study.**

| **Primer name** | **Primer Sequence** |
| --- | --- |
| *E. tracheiphila* IDI pET forward | CACC ATG AAA CCG AGT GAT ACT CTA AGT CAA CGC AAG G |
| *E. tracheiphila* IDI reverse | CAC GGG GAT AAT GCC GGG AGG |
| *R. etli* IDS2 pET forward | CACC ATG ATT TCG AAT CAC CAA GCC GAC GTG G |
| *R. etli* IDS2 pET reverse | CAA AGG CGC GCA ATC CAG CG |
