# Supplementary material for: Unraveling a Tangled Skein: Evolutionary Analysis of the Bacterial Gibberellin Biosynthetic Operon
Source: mSphere. 2020 Jun 3;5(3):e00292-20. doi: 10.1128/mSphere.00292-20 (PMC7273348; doi:10.1128/mSphere.00292-20)
Supplement: FIG S2 [file mSphere.00292-20-sf002.pdf]

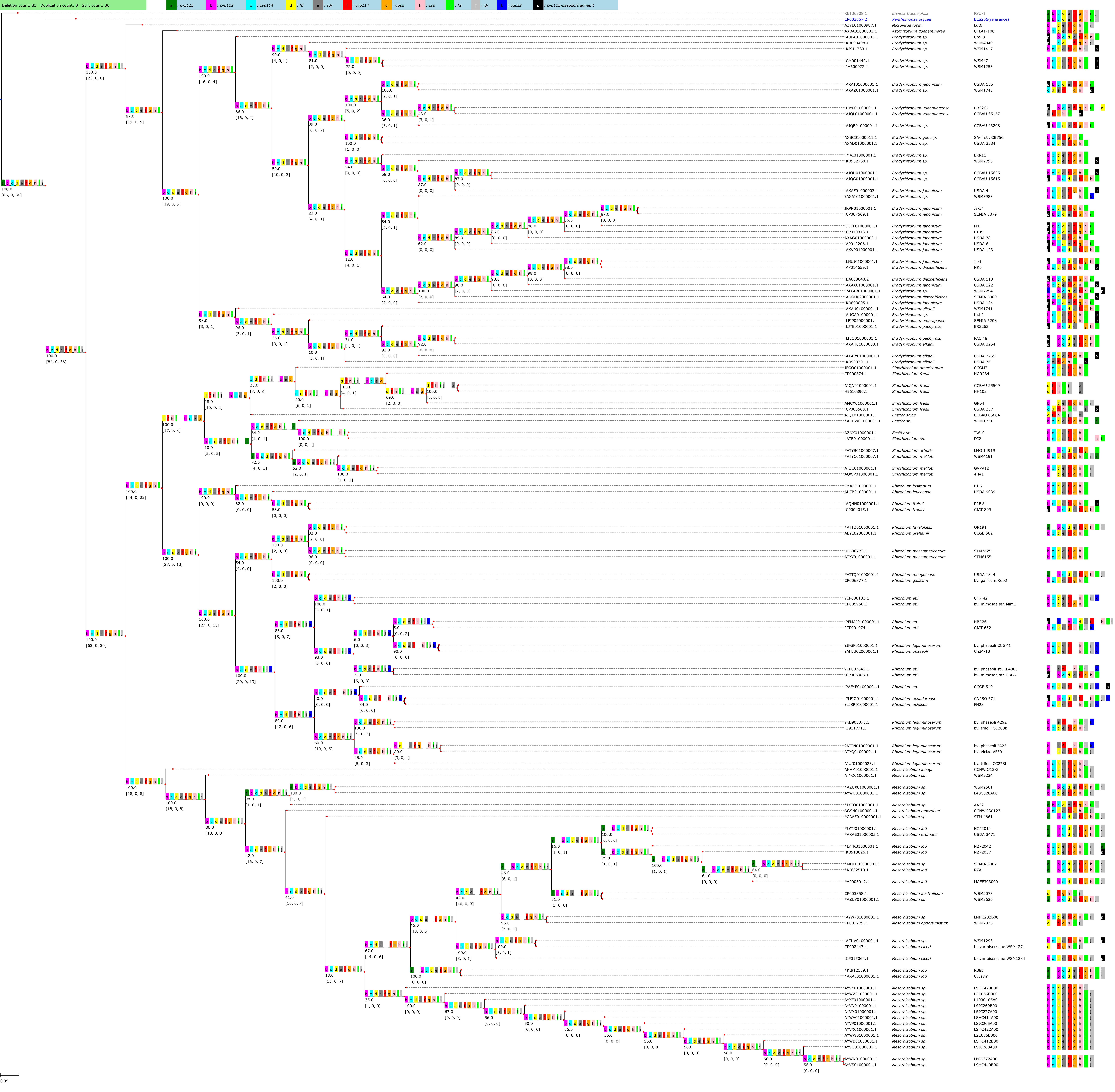

Deletion count: 85 Duplication count: 0 Split count: 36
a : cyp115
b : cyp112
c : cyp114
d : fd
e : sdr
f : cyp117
g : ggps
h : cps
i : ks
j : idi
o : ggps2
p : cyp115-pseudo/fragment
KE136308.1 Erwinia tracheiphila PSU-1
CP003057.2 Xanthomonas oryzae BLS256(reference)
AZYE01000987.1 Microvirga lupini Lut6
AXBA01000001.1 Azorhizobium doebereinerae UFLA1-100
!AUFA01000001.1 Bradyrhizobium sp. CpS.3
!KB890498.1 Bradyrhizobium sp. WSM4349
!KI911783.1 Bradyrhizobium sp. WSM1417
!CM001442.1 Bradyrhizobium sp. WSM471
!JH600072.1 Bradyrhizobium sp. WSM1253
!AXAT01000001.1 Bradyrhizobium japonicum USDA 135
!AXAZ01000001.1 Bradyrhizobium sp. WSM1743
!LJYF01000001.1 Bradyrhizobium yuanmingense BR3267
!AJQL01000001.1 Bradyrhizobium yuanmingense CCBAU 35157
!AJQE01000001.1 Bradyrhizobium sp. CCBAU 43298
AXBC01000011.1 Bradyrhizobium genosp. SA-4 str. CB756
AXAD01000001.1 Bradyrhizobium sp. USDA 3384
FMAI01000001.1 Bradyrhizobium sp. ERR11
!KB902768.1 Bradyrhizobium sp. WSM2793
!AJQH01000001.1 Bradyrhizobium sp. CCBAU 15635
!AJQG01000001.1 Bradyrhizobium sp. CCBAU 15615
!AXAF01000003.1 Bradyrhizobium japonicum USDA 4
?AXAY01000001.1 Bradyrhizobium sp. WSM3983
JRPN01000001.1 Bradyrhizobium japonicum Is-34
!CP007569.1 Bradyrhizobium japonicum SEMIA 5079
!JGCL01000001.1 Bradyrhizobium japonicum FN1
!CP010313.1 Bradyrhizobium japonicum E109
AXAG01000003.1 Bradyrhizobium japonicum USDA 38
!AP012206.1 Bradyrhizobium japonicum USDA 6
!AXVP01000001.1 Bradyrhizobium japonicum USDA 123
!LGUJ01000001.1 Bradyrhizobium japonicum Is-1
!AP014659.1 Bradyrhizobium diazoefficiens NK6
!BA000040.2 Bradyrhizobium diazoefficiens USDA 110
!AXAX01000001.1 Bradyrhizobium japonicum USDA 122
!?AXAB01000001.1 Bradyrhizobium sp. WSM2254
!ADOU02000001.1 Bradyrhizobium diazoefficiens SEMIA 5080
!KB893805.1 Bradyrhizobium japonicum USDA 124
!AXAU01000001.1 Bradyrhizobium elkanii WSM1741
!AUGA01000001.1 Bradyrhizobium sp. th.b2
!LFIP02000001.1 Bradyrhizobium embrapense SEMIA 6208
!LJYE01000001.1 Bradyrhizobium pachyrhizi BR3262
!LFIQ01000001.1 Bradyrhizobium pachyrhizi PAC 48
!AXAH01000003.1 Bradyrhizobium elkanii USDA 3254
!AXAW01000001.1 Bradyrhizobium elkanii USDA 3259
!KB900701.1 Bradyrhizobium elkanii USDA 76
JFGO01000001.1 Sinorhizobium americanum CCGM7
CP000874.1 Sinorhizobium fredii NGR234
AJQN01000001.1 Sinorhizobium fredii CCBAU 25509
HE616890.1 Sinorhizobium fredii HH103
AMCX01000001.1 Sinorhizobium fredii GR64
!CP003563.1 Sinorhizobium fredii USDA 257
AJQT01000001.1 Ensifer sojae CCBAU 05684
*AZUW01000001.1 Ensifer sp. WSM1721
AZNX01000001.1 Ensifer sp. TW10
LATE01000001.1 Sinorhizobium sp. PC2
*ATYB01000007.1 Sinorhizobium arboris LMG 14919
*ATYC01000007.1 Sinorhizobium meliloti WSM4191
ATZC01000001.1 Sinorhizobium meliloti GVPV12
AQWP01000001.1 Sinorhizobium meliloti 4H41
FMAF01000001.1 Rhizobium lusitanum P1-7
AUFB01000001.1 Rhizobium leucaenae USDA 9039
!AQHN01000001.1 Rhizobium freirei PRF 81
!CP004015.1 Rhizobium tropici CIAT 899
*ATTO01000001.1 Rhizobium favelukesii OR191
AEYE02000001.1 Rhizobium grahamii CCGE 502
HF536772.1 Rhizobium mesoamericanum STM3625
ATYY01000001.1 Rhizobium mesoamericanum STM6155
*ATTQ01000001.1 Rhizobium mongolense USDA 1844
CP006877.1 Rhizobium gallicum bv. gallicum R602
?CP000133.1 Rhizobium etli CFN 42
CP005950.1 Rhizobium etli bv. mimosae str. Mim1
!?FMAJ01000001.1 Rhizobium sp. HBR26
?CP001074.1 Rhizobium etli CIAT 652
?JFGP01000001.1 Rhizobium leguminosarum bv. phaseoli CCGM1
?AHJU02000001.1 Rhizobium phaseoli Ch24-10
?CP007641.1 Rhizobium etli bv. phaseoli str. IE4803
!CP006986.1 Rhizobium etli bv. mimosae str. IE4771
!?AEYF01000001.1 Rhizobium sp. CCGE 510
!?LFIO01000001.1 Rhizobium ecuadorense CNPSO 671
?LJSR01000001.1 Rhizobium acidisoli FH23
?KB905373.1 Rhizobium leguminosarum bv. phaseoli 4292
KI911771.1 Rhizobium leguminosarum bv. trifolii CC283b
?ATTN01000001.1 Rhizobium leguminosarum bv. phaseoli FA23
ATYQ01000001.1 Rhizobium leguminosarum bv. viciae VF39
AJUI01000023.1 Rhizobium leguminosarum bv. trifolii CC278f
AHAM01000001.1 Mesorhizobium alhagi CCNWXJ12-2
ATYO01000001.1 Mesorhizobium sp. WSM3224
*AZUX01000001.1 Mesorhizobium sp. WSM2561
AYWU01000001.1 Mesorhizobium sp. L48C026A00
*LYTO01000001.1 Mesorhizobium sp. AA22
AGSN01000001.1 Mesorhizobium amorphae CCNWGS0123
*CAAF010000001.1 Mesorhizobium sp. STM 4661
*LYTJ01000001.1 Mesorhizobium loti NZP2014
*AXAE01000005.1 Mesorhizobium erdmanii USDA 3471
*LYTK01000001.1 Mesorhizobium loti NZP2042
!KB913026.1 Mesorhizobium loti NZP2037
*MDLH01000001.1 Mesorhizobium sp. SEMIA 3007
*KI632510.1 Mesorhizobium loti R7A
*AP003017.1 Mesorhizobium loti MAFF303099
CP003358.1 Mesorhizobium australicum WSM2073
*AZUY01000001.1 Mesorhizobium sp. WSM3626
!AYWP01000001.1 Mesorhizobium sp. LNHC232B00
CP002279.1 Mesorhizobium opportunistum WSM2075
!AZUV01000001.1 Mesorhizobium sp. WSM1293
CP002447.1 Mesorhizobium ciceri biovar biserrulae WSM1271
!CP015064.1 Mesorhizobium ciceri biovar biserrulae WSM1284
*KI912159.1 Mesorhizobium loti R88b
*AXAL01000001.1 Mesorhizobium loti CJ3sym
AYVY01000001.1 Mesorhizobium sp. LSHC420B00
AYWZ01000001.1 Mesorhizobium sp. L2C066B000
AYXF01000001.1 Mesorhizobium sp. L103C105A0
AYVN01000001.1 Mesorhizobium sp. LSJC269B00
AYVM01000001.1 Mesorhizobium sp. LSJC277A00
AYWA01000001.1 Mesorhizobium sp. LSHC414A00
AYVP01000001.1 Mesorhizobium sp. LSJC265A00
AYVX01000001.1 Mesorhizobium sp. LSHC422A00
AYWW01000001.1 Mesorhizobium sp. L2C085B000
AYWB01000001.1 Mesorhizobium sp. LSHC412B00
AYVO01000001.1 Mesorhizobium sp. LSJC268A00
AYWN01000001.1 Mesorhizobium sp. LNJC372A00
AYVS01000001.1 Mesorhizobium sp. LSHC440B00
0.09
