# Supplementary material for: Unraveling a Tangled Skein: Evolutionary Analysis of the Bacterial Gibberellin Biosynthetic Operon
Source: mSphere. 2020 Jun 3;5(3):e00292-20. doi: 10.1128/mSphere.00292-20 (PMC7273348; doi:10.1128/mSphere.00292-20)
Supplement: FIG S3 [file mSphere.00292-20-sf003.pdf]

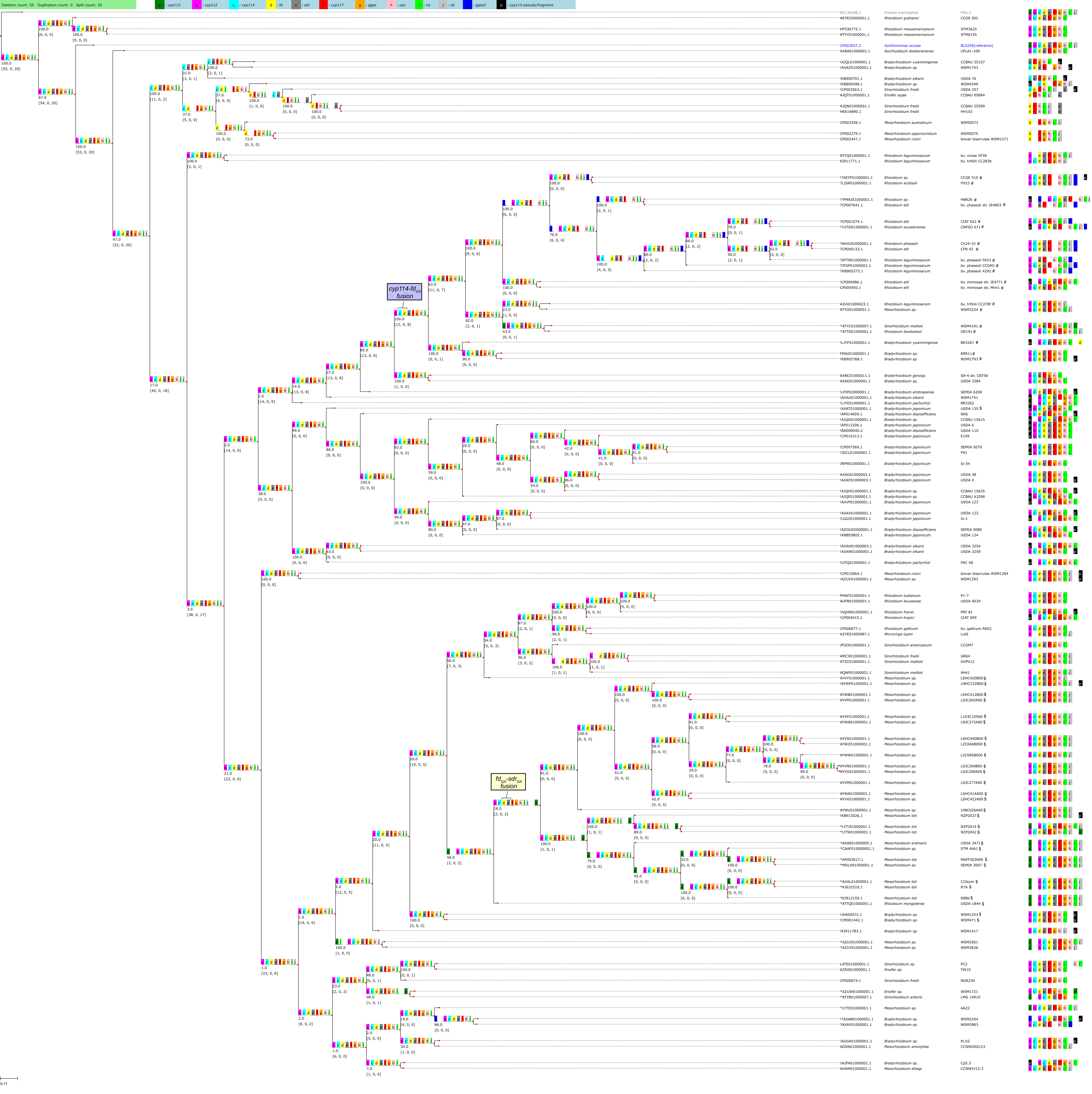

Deletion count: 55 Duplication count: 0 Split count: 20
a : cyp115
b : cyp112
c : cyp114
d : fd
e : sdr
f : cyp117
g : ggps
h : cps
i : ks
j : idi
k : ggps2
p : cyp115-pseudo/fragment
cyp114-fdGA fusion
fdGA-sdrGA fusion
0.11
